# Forkhead O Transcription Factor 4 Restricts HBV Covalently Closed Circular DNA Transcription and HBV Replication through Genetic Downregulation of Hepatocyte Nuclear Factor 4 Alpha and Epigenetic Suppression of Covalently Closed Circular DNA via Interacting with Promyelocytic Leukemia Protein

**DOI:** 10.1128/jvi.00546-22

**Published:** 2022-06-13

**Authors:** Yuqi Li, Minjing He, Ruijie Gong, Ziteng Wang, Lin Lu, Shu Peng, Zhiyun Duan, Ying Feng, Yi Liu, Bo Gao

**Affiliations:** a Department of Immunology, School of Basic Medical Sciences, Shanghai Medical College of Fudan University, and Liver Cancer Institute, Zhongshan Hospital, Fudan University, Shanghai, People’s Republic of China; b Department of Digestive Diseases, Huashan Hospital, Fudan University, Shanghai, People’s Republic of China; University of Southern California

**Keywords:** FoxO4, HBV cccDNA, HNF4α, PML, epigenetic modification

## Abstract

Nuclear located hepatitis B virus (HBV) covalently closed circular DNA (cccDNA) remains the key obstacle to cure chronic hepatitis B (CHB). In our previous investigation, it was found that FoxO4 could inhibit HBV core promoter activity through downregulating the expression of HNF4α. However, the exact mechanisms whereby FoxO4 inhibits HBV replication, especially its effect on cccDNA, remain unclear. Here, our data further revealed that FoxO4 could effectively inhibit cccDNA mediated transcription and HBV replication without affecting cccDNA level. Mechanistic study showed that FoxO4 could cause epigenetic suppression of cccDNA. Although FoxO4-mediated downregulation of HNF4α contributed to inhibiting HBV core promoter activity, it had little effect on cccDNA epigenetic regulation. Further, it was found that FoxO4 could colocalize within promyelocytic leukemia protein (PML) nuclear bodies and interact with PML. Of note, PML was revealed to be critical for FoxO4-mediated inhibition of cccDNA epigenetic modification and of the following cccDNA transcription and HBV replication. Furthermore, FoxO4 was found to be downregulated in HBV-infected hepatocytes and human liver tissues, and it was negatively correlated with cccDNA transcriptional activity in CHB patients. Together, these findings highlight the role of FoxO4 in suppressing cccDNA transcription and HBV replication via genetic downregulation of HNF4α and epigenetic suppression of cccDNA through interacting with PML. Targeting FoxO4 may present as a new therapeutic strategy against chronic HBV infection.

**IMPORTANCE** HBV cccDNA is a determining factor for viral persistence and the main obstacle for a cure of chronic hepatitis B. Strategies that target cccDNA directly are therefore of great importance in controlling persistent HBV infection. In present investigation, we found that FoxO4 could efficiently suppress cccDNA transcription and HBV replication without affecting the level of cccDNA itself. Further, our data revealed that FoxO4 might inhibit cccDNA function via a two-part mechanism: one is to epigenetically suppress cccDNA transcription via interacting with PML, and the other is to inhibit HBV core promoter activity via the genetic downregulation of HNF4α. Of note, HBV might dampen the expression of FoxO4 for its own persistent infection. We propose that manipulation of FoxO4 may present as a potential therapeutic strategy against chronic HBV infection.

## INTRODUCTION

Hepatitis B virus (HBV) infection remains still a major public health burden globally. Although effective prophylactic vaccines have been available since decades, around 260 million people worldwide suffer from chronic HBV infection, which may develop into liver cirrhosis, and even hepatocellular carcinoma (HCC), causing nearly 1 million deaths each year (1, 2).

HBV covalently closed circular DNA (cccDNA), the template for transcription of pregenomic RNA (pgRNA) and other HBV RNAs, accumulates as a stable episomal form of the viral genome decorated with host histones and nonhistone proteins. Due to its high stability in the nuclei of infected liver cells, cccDNA is considered to be the determining factor of viral persistence and the main obstacle to the treatment of chronic hepatitis B (CHB) (3, 4). Currently, there are two main antiviral drugs for CHB, i.e., alpha interferon (IFN-α) and nucleos(t)ide analogues (NAs). The usage of IFN-α in treating CHB is limited by its systemic side effects, while treatment with NAs needs to be maintained without cessation to avoid HBV relapse because NAs act on the cytoplasm-located HBV polymerase which possesses reverse transcriptase (RT) activity but not act directly on the nuclear viral cccDNA (5, 6). Therefore, further identification of factors that target cccDNA directly is of great importance for the development of new therapeutic strategy against persistent HBV infection.

Currently, there are several strategies that might target HBV cccDNA directly. Reports indicate that sequence-specific RNA-guided nucleases (RGNs), including transcription activator-like effector nucleases (TALENs) and clustered regularly interspaced short palindromic repeats (CRISPR) systems, could target and eradicate nuclear cccDNA. However, challenges, such as the delivery of RGNs into the nuclei of infected cells, the specificity of RGNs, and the DNA damage response triggered during the gene-editing process, remain to be addressed (6, 7). It is also reported that immune-mediated clearance of partial cccDNA could be obtained by IFN-α, lymphotoxin-β receptor (LTβR) agonist or tumor necrosis factor alpha (TNF-α) through upregulating the expression of APOBEC3A/3B cytidine deaminase, which could deaminate the cccDNA in the nuclei of infected cells and thus lead to the degradation of cccDNA (6). However, these immune-mediated therapies could not eliminate cccDNA completely since cccDNA may exist in distinct forms that differ in methylation, chromatinization, and some other properties (3, 7). Accumulating evidence indicates that epigenetic regulation can regulate cccDNA transcriptional activity directly and subsequently control HBV replication, and epigenetic silencing of HBV cccDNA has been proposed to be a more feasible and practical way to control HBV infection (8–10).

Forkhead box O (FoxO) family of transcription factors comprise a highly conserved forkhead box domain (also called winged helix domain), which can bind directly to various target DNA sequences in nuclei (11). In the human genome, FoxO family members mainly include FoxO1, FoxO3, FoxO4, and FoxO6. FoxO1, FoxO3, and FoxO4 are widely expressed in various tissues, whereas the expression of FoxO6 is mainly limited to the adult brain. FoxOs participate in the regulation of diverse cellular processes, such as cell cycle, apoptosis, differentiation, proliferation, energy metabolism, and autophagy, and pose as therapeutic targets for a variety of diseases, including cancer, aging diseases, and viral infection (12–17). In terms of HBV infection, it is reported that FoxO1 can promote the transcription and replication of HBV (18). Our previous study revealed that HBV could significantly downregulate the expression of FoxO4, but not FoxO3 and FoxO1, and that FoxO4 was revealed to inhibit the HBV core promoter activity through ERK1/2-HNF4α axis (19). Consistent with our investigation, Fu et al. (20) also reported that HBV appeared to suppress FoxO4 expression, and FoxO4 displayed an inhibitory effect on HBV replication. However, the exact mechanisms whereby FoxO4 inhibits HBV replication, especially its effect on cccDNA, remain unclear.

Here, our data showed that FoxO4 suppressed cccDNA-driven transcription and HBV replication without affecting the level of cccDNA itself. Mechanistically, besides its inhibition of HBV core promoter through downregulation of HNF4α, FoxO4 exerted epigenetic suppression on HBV cccDNA via colocalizing with promyelocytic leukemia protein nuclear bodies (PML-NB) and interacting with PML. Together, our data demonstrated that FoxO4 could suppress cccDNA transcription and HBV replication through genetic downregulation of HNF4α and epigenetic silencing of cccDNA though targeting PML bodies.

## RESULTS

### FoxO4 overexpression restricts HBV cccDNA transcription and HBV replication.

**FIG 1:**
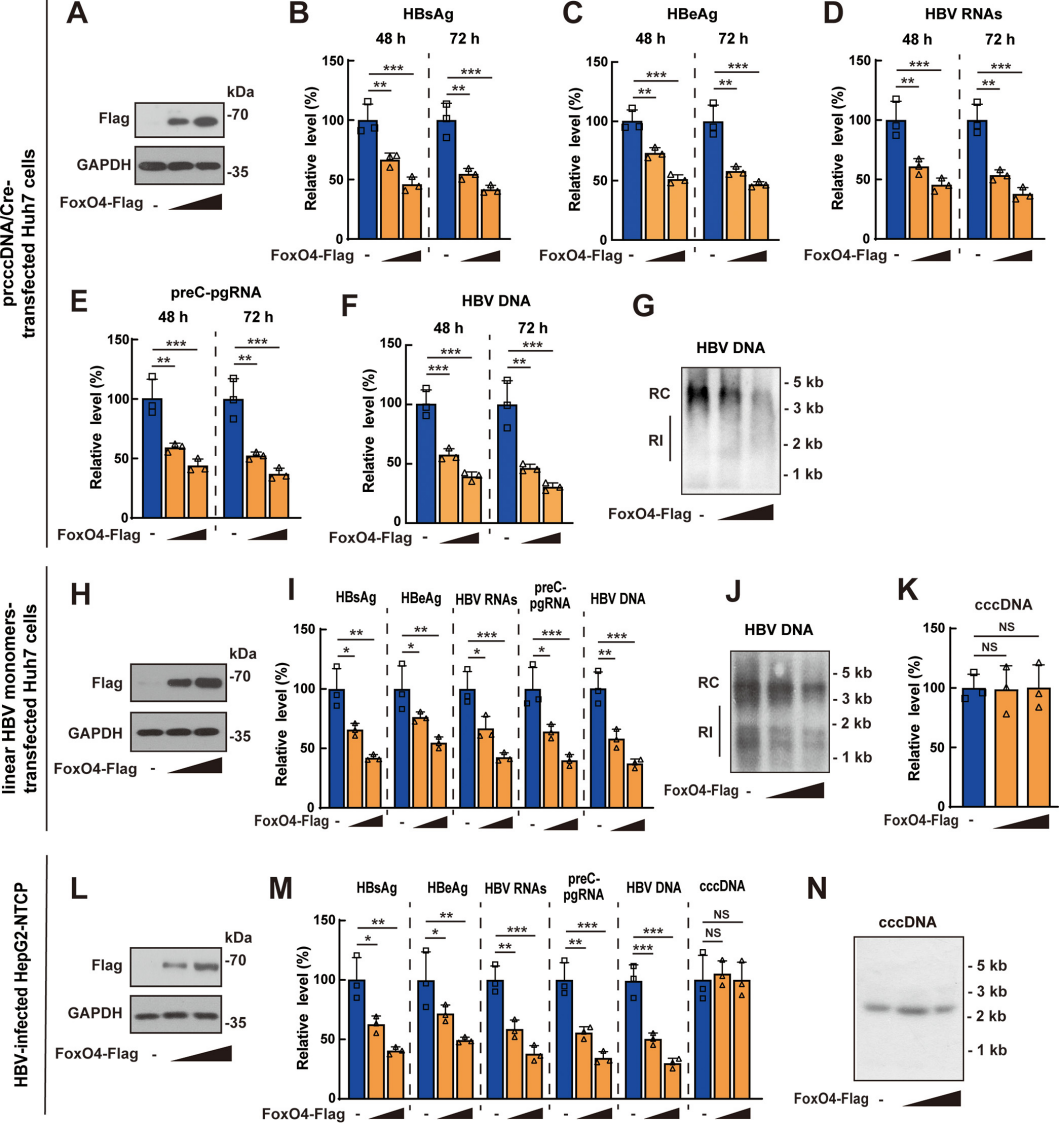
FoxO4 overexpression inhibits cccDNA-mediated transcription and HBV replication. (A to G) PrcccDNA/Cre was transfected into Huh7 cells together with control empty vector or increasing amount of FoxO4-Flag for 48 or 72 h. (A) The protein level of FoxO4-Flag in prcccDNA/Cre-transfected Huh7 cells was determined by Western blotting. The levels of HBsAg (B), HBeAg (C), HBV RNAs (D), and preC-pgRNA (E) were determined by ELISA and qRT-PCR, respectively. The levels of HBV DNA were determined by qPCR (F) and Southern blotting (G). (H to K) Linear HBV monomers were transfected into Huh7 cells together with control empty vector or increasing amounts of FoxO4-Flag for 48 h, and the levels of FoxO4-Flag, HBsAg, HBeAg, preC-pgRNA, and HBV DNA were determined as in panels A to G. cccDNA was extracted by Hirt’s method as described in Materials and Methods and then subjected to quantification by qPCR (K). (L to N) HepG2-NTCP cells were electrotransfected with FoxO4-Flag for 48 h and then infected with HBV at 10^3^ vge/cell as described in Materials and Methods. The levels of FoxO4-Flag, cccDNA (at day 3 postinfection) and the levels of HBsAg, HBeAg, preC-pgRNA, and HBV DNA (at day 9 postinfection) were determined as described above. The data show means ± the SD of triplicates and are representative of three independent experiments (*, *P < *0.05; **, *P < *0.01; ***, *P < *0.001; NS, no significance; RI, replicative intermediates).

To investigate the effect of FoxO4 on HBV cccDNA, precursor plasmid cccDNA (prcccDNA) plus Cre-recombinase expression plasmid (pCMV-Cre) were transfected into Huh7 cells together with Flag-tagged FoxO4 expression plasmid (FoxO4-Flag) or control empty vector, followed by the evaluation of HBV transcription and replication at indicated time points. Western blotting verified the efficient expression of exogenous FoxO4 (Fig. 1A). Enzyme-linked immunosorbent assay (ELISA) results showed that FoxO4 overexpression could significantly decrease the levels of HBsAg and HBeAg (Fig. 1B and C). Data from qRT-PCR showed that overexpression of FoxO4 effectively downregulated the levels of HBV RNAs (Fig. 1D) and preC-pgRNA (Fig. 1E). Both qPCR and Southern blotting demonstrated that overexpression of FoxO4 downregulated the level of HBV DNA effectively (Fig. 1F and G). Considering that rcccDNA might not fully recapitulate the characteristics of *bona fide* cccDNA due to the existence of the chimeric intron sequence (21), we used another well-established cccDNA cell model by transfecting the Huh7 cells with linear HBV monomers, as described by Pollicino et al. (22), and then tested the effect of FoxO4 on cccDNA. Consistent with data obtained from rcccDNA model, FoxO4 overexpression significantly downregulated the levels of HBV proteins, HBV transcripts, and HBV DNA in linear HBV monomer-transfected Huh7 cells (Fig. 1H to J); however, overexpression of FoxO4 did not display significant effect on the level of cccDNA itself (Fig. 1K). Further, we examined the effect of FoxO4 overexpression on cccDNA in HBV-infected HepG2-NTCP cells and found that FoxO4 significantly inhibited cccDNA transcription and HBV replication without affecting the level of cccDNA itself (Fig. 1L to N). We had also investigated the effect of FoxO4 on cccDNA in a rcccDNA mouse model by injecting prcccDNA/Cre into C57BL/6 mice together with FoxO4-Flag or control empty vector using hydrodynamic gene transfer technique. Consistent with the *in vitro* data, FoxO4 expression could also significantly inhibit rcccDNA-driven transcription and HBV replication in mice (see Fig. S1A to G in the supplemental material).

### Downregulation of FoxO4 enhances HBV cccDNA transcription and HBV replication.

**FIG 2:**
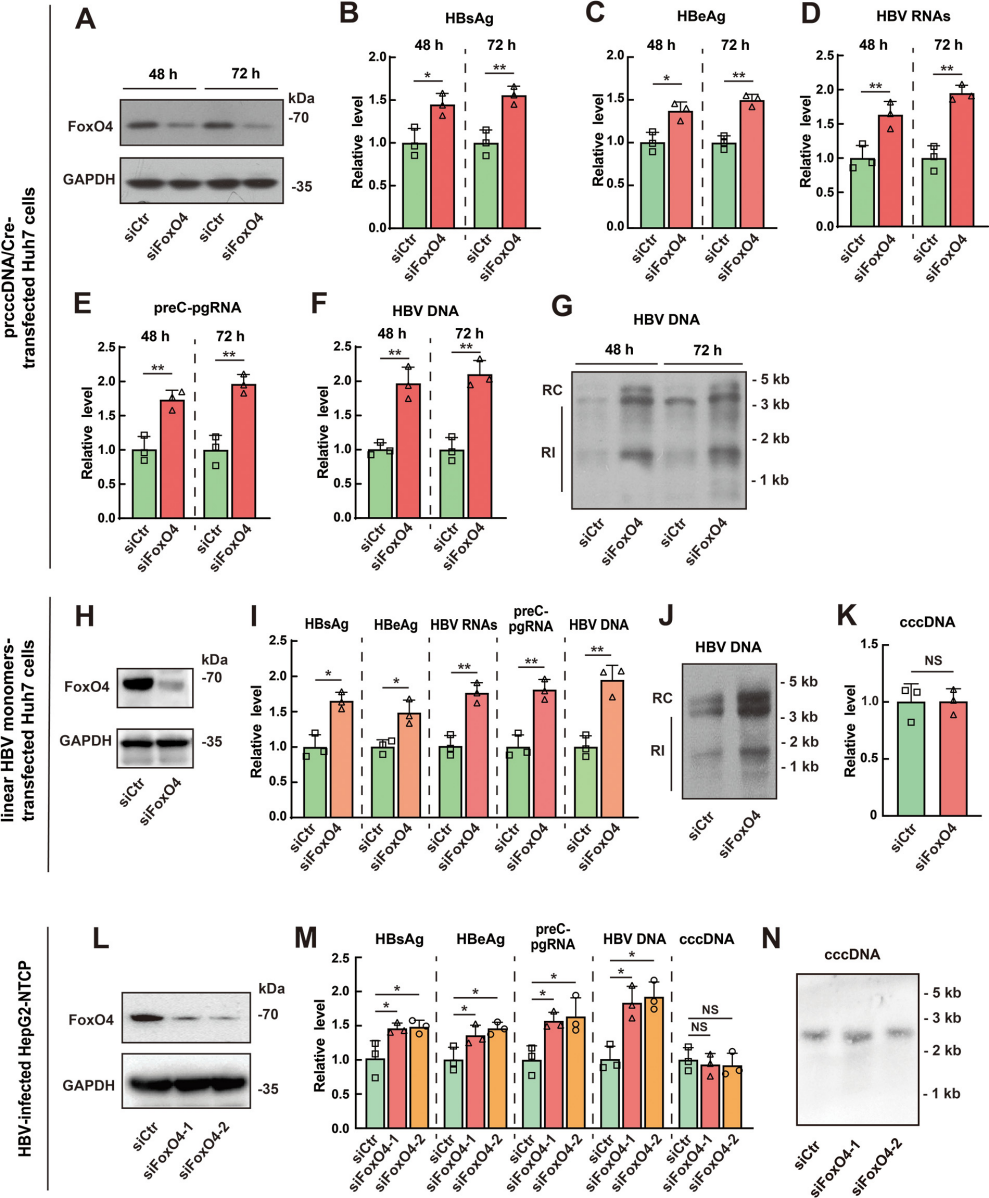
FoxO4 downregulation enhances cccDNA-mediated transcription and HBV replication. (A to G) Huh7 cells were transfected with control or FoxO4 siRNA for 48 h, and cells were then further transfected with prcccDNA/Cre for another 48 h. (A) The level of FoxO4-Flag was determined by Western blotting. The levels of HBsAg (B) and HBeAg (C) were detected by ELISA, and the levels of HBV RNAs (D) and preC-pgRNA (E) were determined by qRT-PCR. The levels of HBV DNA were determined by qPCR (F) and Southern blotting (G). (H to K) Huh7 cells were transfected with control or FoxO4 siRNA for 48 h, and cells were then further transfected with linear HBV monomers for another 48 h. The levels of exogenous FoxO4 (H), HBV proteins, transcripts, and HBV DNA (I and J) were determined as in panels A to G, and the level of cccDNA was determined by qPCR (K). (L to N) HepG2-NTCP cells were electrotransfected with control or FoxO4-specific siRNA for 48 h and then infected with HBV at 10^3^ vge/cell. The levels of FoxO4-Flag, cccDNA (at day 3 postinfection) and the levels of HBsAg, HBeAg, HBV RNAs, preC-pgRNA, and HBV DNA (at day 9 postinfection) were determined as described above. The data are shown as means ± the SD of triplicates and are representative of three independent experiments (*, *P < *0.05; **, *P < *0.01; NS, no significance; RI, replicative intermediates).

To further confirm the inhibitory effect of FoxO4 on HBV cccDNA-mediated transcription and HBV replication, we downregulated the expression of FoxO4 in Huh7 cells via siRNA technique (Fig. 2A), and then transfected the cells with prcccDNA plus pCMV-Cre (prcccDNA/Cre). We found that downregulation of FoxO4 significantly enhanced the rcccDNA-mediated transcription and HBV replication, as demonstrated by the increased levels of HBsAg (Fig. 2B), HBeAg (Fig. 2C), HBV RNAs (Fig. 2D), preC-pgRNA (Fig. 2E), and HBV DNA (Fig. 2F and G). Further, we examined the effect of FoxO4 downregulation on cccDNA transcription and HBV production in linear HBV monomers-transfected Huh7 cells and found that small interfering RNA (siRNA)-mediated downregulation of FoxO4 could significantly upregulate the levels of HBV proteins, transcripts, and HBV DNA (Fig. 2H to J) but had little effect on the level of HBV cccDNA in this cell model (Fig. 2K). Furthermore, we investigated the effect of FoxO4 knockdown on cccDNA in an HBV-infected HepG2-NTCP cell model. Consistent with the data obtained from prcccDNA/Cre- or linear HBV monomers-transfected Huh7 cells, FoxO4 downregulation significantly enhanced cccDNA-driven transcription and HBV replication but had no significant effect on the level of cccDNA itself in HBV-infected HepG2-NTCP cells (Fig. 2L to N).

Taken together, these data demonstrated that FoxO4 could repress cccDNA-mediated transcription and HBV replication, while it did not have significant effect on the level of cccDNA itself.

### FoxO4 promotes epigenetic suppression of HBV cccDNA by promoting its heterochromatinization.

**FIG 3:**
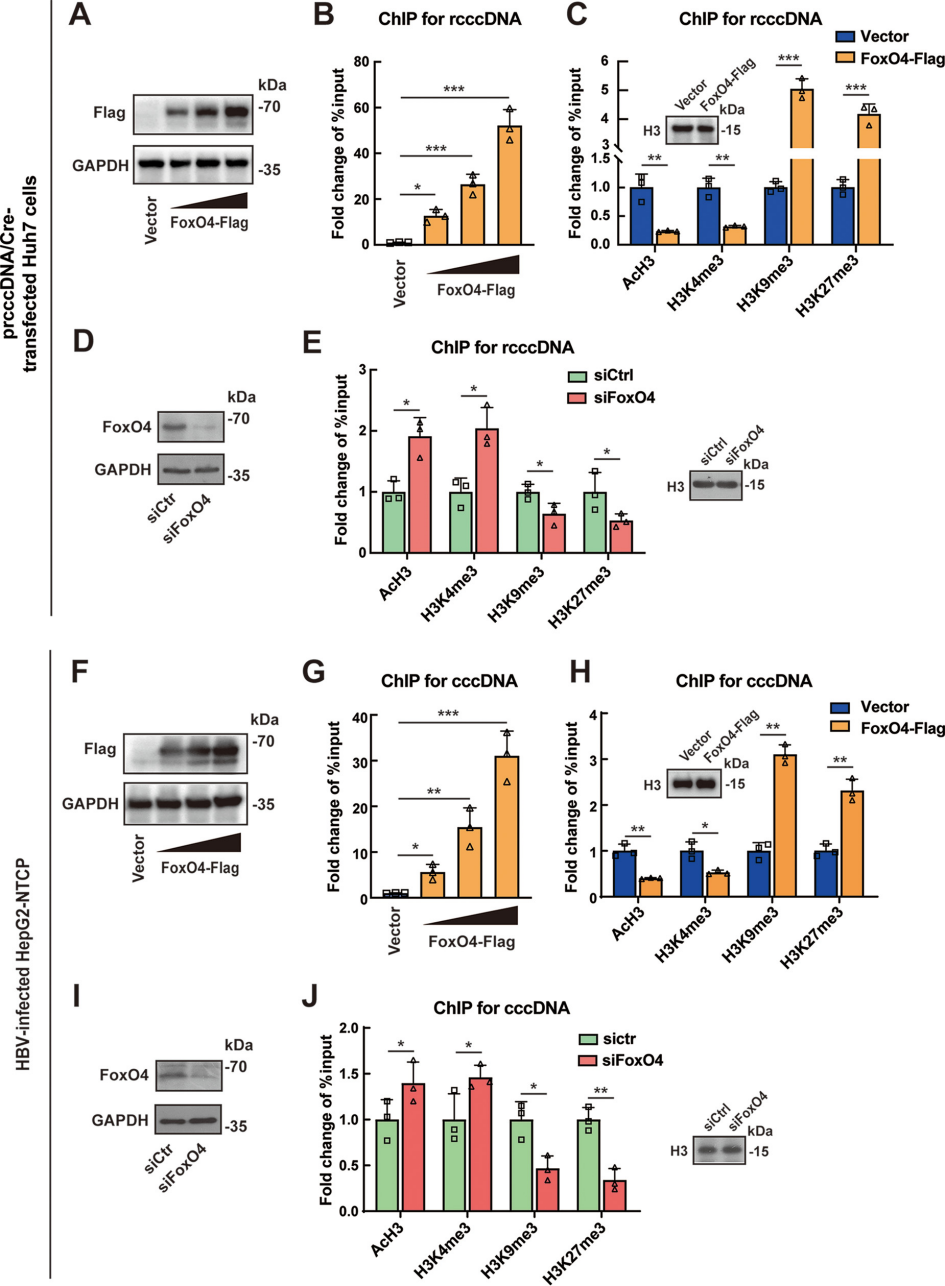
FoxO4 promotes epigenetic suppression of HBV cccDNA. (A) prcccDNA/Cre was transfected into Huh7 cells together with control empty vector or increasing amount of FoxO4-Flag for 48 h. The levels of FoxO4-Flag were determined by Western blotting. (B) Cells were treated as in panel A, and the binding of increasing doses of FoxO4-Flag to rcccDNA was determined by ChIP assay. The data are shown as the fold change to control empty vector-transfected cells after being normalized to input and control IgG. The value obtained from empty vector-transfected cells was set to 1. (C) The effect of FoxO4 overexpression on the recruitment of AcH3, H3K4me3, H3K9me3, and H3K27me3 onto rcccDNA was determined by ChIP assay as in panel B. (D) Huh7 cells were transfected with control or FoxO4 siRNA for 48 h, and cells were then further transfected with prcccDNA/Cre for another 48 h. The knockdown efficiency of FoxO4 was determined by Western blotting. (E) Cells were treated as in panel D, and the effect of FoxO4 downregulation on the recruitment of AcH3, H3K4me3, H3K9me3, and H3K27me3 onto rcccDNA was determined by ChIP assay. The data are shown as the fold change to control siRNA-transfected cells after being normalized to input and control IgG. (F) HepG2-NTCP cells were electrotransfected with FoxO4-Flag for 48 h and then infected with HBV. At 3 days postinfection, the level of FoxO4-Flag was determined by Western blotting. (G) Cells were treated as in panel F, the binding of FoxO4-Flag to cccDNA was determined by ChIP assays. The data are shown as the fold change versus control empty vector-transfected cells after normalized to input and control IgG. (H) The effect of FoxO4 on the recruitment of AcH3, H3K4me3, H3K9me3, and H3K27me3 onto cccDNA was determined by ChIP assays as in panel G. (I) HepG2-NTCP cells were electrotransfected with control or FoxO4-specific siRNA for 48 h and then infected with HBV at 10^3^ vge/cell. At 3 days postinfection, the level of endogenous FoxO4 protein was detected by Western blotting. (J) Cells were treated as in panel I, and the effect of FoxO4 on the recruitment of AcH3, H3K4me3, H3K9me3, and H3K27me3 onto cccDNA was determined by ChIP assays. The data are shown as the fold change to control siRNA-transfected cells after being normalized to input and control IgG. The data are shown as means ± the SD of triplicates and are representative of three independent experiments (*, *P < *0.05; **, *P < *0.01; ***, *P < *0.001).

The data presented above showed that FoxO4 significantly inhibited cccDNA-mediated transcription and HBV replication but had little impact on the level of cccDNA itself, indicating that FoxO4 might be of relevance to the silence of cccDNA function. It is now evident that epigenetic modifications play a pivotal role in the transcription of nuclear-located cccDNA (10), and recent reports indicate that FoxO proteins primarily function as transcription factors in the nucleus and may act as epigenetic effectors (23, 24); we are thus interested in the effect of FoxO4 on the epigenetic modification of HBV cccDNA. As shown in Fig. 3A to C, FoxO4 could bind to the rcccDNA in a dose-dependent manner, and FoxO4 overexpression could efficiently decrease the recruitment of euchromatin markers (acetylated histone H3 [AcH3] and trimethylation of lysine 4 on histone H3 [H3K4me3]) to rcccDNA but increase the recruitment of heterochromatin markers (trimethylation of lysine 9 on histone H3 [H3K9me3] and trimethylation of lysine 27 on histone H3 [H3K27me3]) to rcccDNA in prcccDNA/Cre-transfected Huh7 cells. Next, we knocked down the expression of FoxO4 by using an siRNA technique in Huh7 cells (Fig. 3D) and then examined the epigenetic markers of rcccDNA. Our data showed that FoxO4 downregulation could enhance the recruitment of euchromatin markers AcH3 and H3K4me3 to rcccDNA but reduce the recruitment of heterochromatin markers H3K9me3 and H3K27me3 to rcccDNA (Fig. 3E). Similar data were obtained in rcccDNA mice (see Fig. S2). Further, we investigated the effect of FoxO4 on the epigenetic regulation of cccDNA in HBV-infected HepG2-NTCP cells. Consistent with the rcccDNA system, FoxO4 overexpression led to the heterochromatination of cccDNA, as demonstrated by the concomitant decrease of AcH3 and H3K4me3 and increase of H3K9me3 and H3K27me3 on cccDNA (Fig. 3F to H), and FoxO4 downregulation led to the euchromatination of cccDNA, as demonstrated by the concomitant increase of AcH3 and H3K4me3 and decrease of H3K9me3 and H3K27me3 on cccDNA in HBV-infected HepG2-NTCP cells (Fig. 3I and J). Taken together, all these data indicated that FoxO4 could bind to cccDNA and lead to the epigenetic suppression of cccDNA through promoting its heterochromatinization.

In our previous investigation, we demonstrated that nuclear-located DNA sensor IFI16 could inhibit HBV cccDNA transcription by integrating innate immune activation and epigenetic suppression (8); we had therefore been interested in whether IFI16 was implicated in FoxO4-mediated anti-HBV activities. However, our data showed that FoxO4 overexpression failed to upregulate the expression level of IFI16 (see Fig. S3A and B); although IFI16 could significantly activate innate immune signaling in rcccDNA cell model, FoxO4 failed to do so (see Fig. S3C and D). Further, knockdown of IFI16 appeared not to have significant effect on rcccDNA-driven transcription and HBV replication (see Fig. S3E and F), indicating that IFI16 might not be involved in FoxO4-mediated anti-cccDNA activity.

### FoxO4-mediated epigenetic suppression of HBV cccDNA is not due to its downregulation of HNF4α.

**FIG 4:**
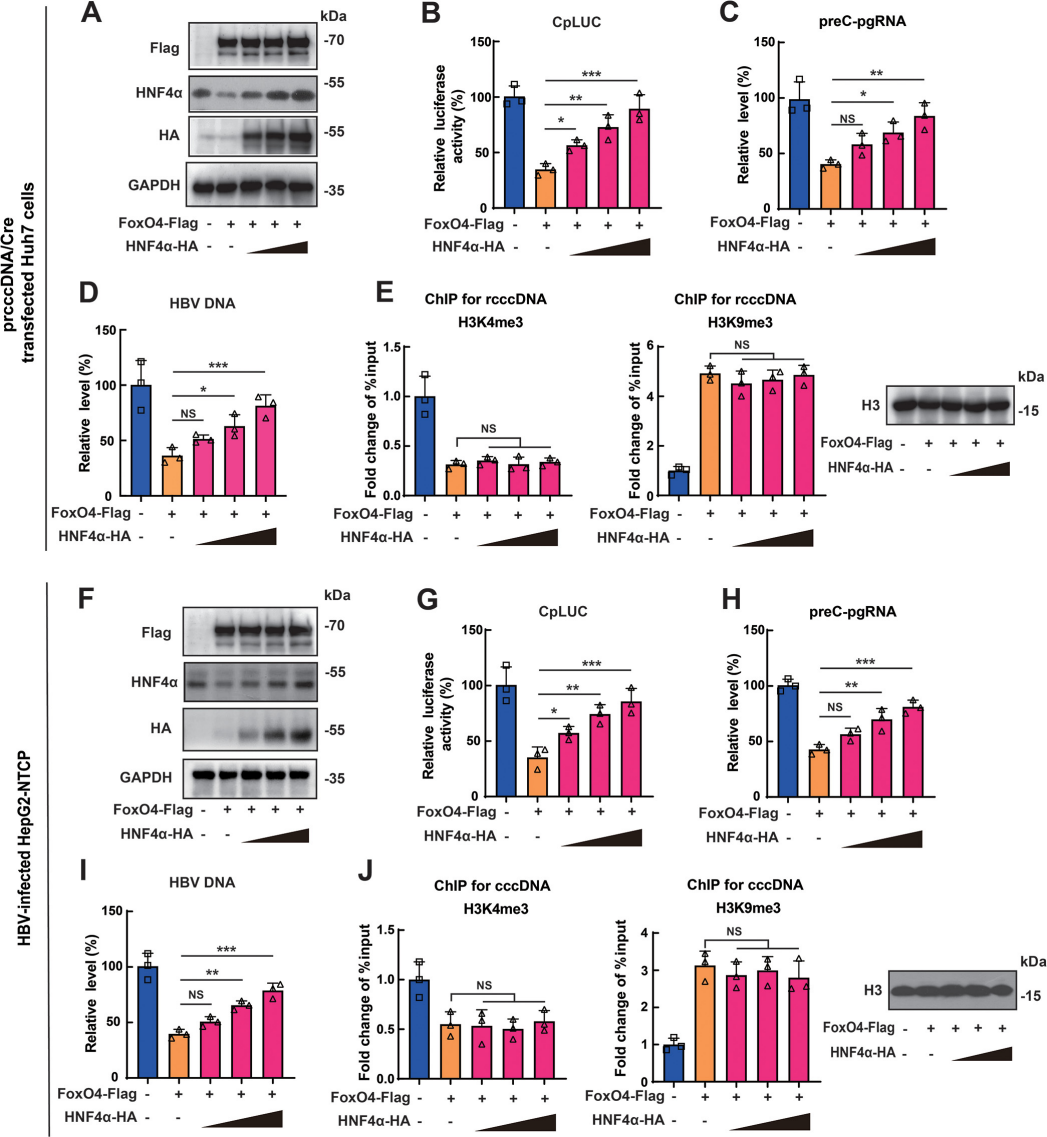
FoxO4-mediated epigenetic suppression of HBV cccDNA is not due to its effect on HNF4α expression. (A and B) The HBV core promoter-dependent reporter plasmid (CpLUC) was transfected into Huh7 cells together with FoxO4-Flag in the absence or presence of increasing doses of HNF4α-HA. At 48 h posttransfection, the protein levels of FoxO4 and HNF4α were determined by Western blotting with the indicated antibodies (A), and the HBV core promoter activity was determined by luciferase assay (B). (C and D) prcccDNA/Cre was transfected into Huh7 cells together with FoxO4-Flag in the absence or presence of increasing doses of HNF4α-HA. At 48 h posttransfection, the levels of preC-pgRNA (C) and HBV DNA (D) were detected by qRT-PCR and qPCR, respectively. (E) Cells were treated as in panels C and D, and the effect of FoxO4 on the recruitment of H3K4me3 and H3K9me3 onto rcccDNA was determined by ChIP assays. The data are shown as the fold change to empty vector-transfected cells after normalized to input and control IgG. (F and G) CpLUC and FoxO4-Flag were cotransfected into HepG2-NTCP cells in the absence or presence of increasing doses of HNF4α-HA. At 48 h posttransfection, the protein levels of FoxO4 and HNF4α were determined by Western blotting with the indicated antibodies (F), and the HBV core promoter activity was determined by a luciferase assay (G). (H to J) FoxO4-Flag was transfected into HepG2-NTCP cells in the absence or presence of increasing doses of HNF4α-HA. at 48 h posttransfection, the cells were then infected with HBV at 10^3^ vge/cell. At day 9 postinfection, levels of preC-pgRNA (H) and HBV DNA (I) were detected by qRT-PCR and qPCR, respectively, and at day 3 postinfection, the effect of FoxO4 on the recruitment of H3K4me3, H3K9me3 to cccDNA was determined by ChIP assay (J). The data are shown as means ± the SD of triplicates and are representative of three independent experiments (*, *P < *0.05; **, *P < *0.01; ***, *P < *0.001; NS, no significance).

Our previous work showed that FoxO4 inhibited HBV core promoter through the downregulation of HNF4α (19), and several reports suggested that HNF4α might be involved in epigenetic regulation of target genes (25, 26). We therefore explored whether HNF4α participated in the FoxO4-mediated epigenetic suppression of HBV cccDNA. Our results showed that FoxO4 overexpression significantly downregulated the expression level of HNF4α in Huh7 cells, whereas HNF4α could significantly reverse the inhibitory effect of FoxO4 on HBV core promoter activity (Fig. 4A and B) and contribute to the reversion of FoxO4-mediated inhibition of rcccDNA transcription and HBV replication (Fig. 4C and D), findings which were in agreement with our previous investigation (19). However, parallel ChIP assays showed that HNF4α expression had little effect on the FoxO4-mediated epigenetic repression of rcccDNA (Fig. 4E). We also tested the effect of HNF4α expression on FoxO4-mediated epigenetic regulation on cccDNA in HBV-infected HepG2-NTCP cells, and data similar to those from the rcccDNA cell model were obtained (Fig. 4F to J). Taken together, these data indicated that HNF4α was not involved in FoxO4-mediated epigenetic suppression of HBV cccDNA.

### FoxO4 colocalizes with PML in nuclear bodies and interacts with PML.

**FIG 5:**
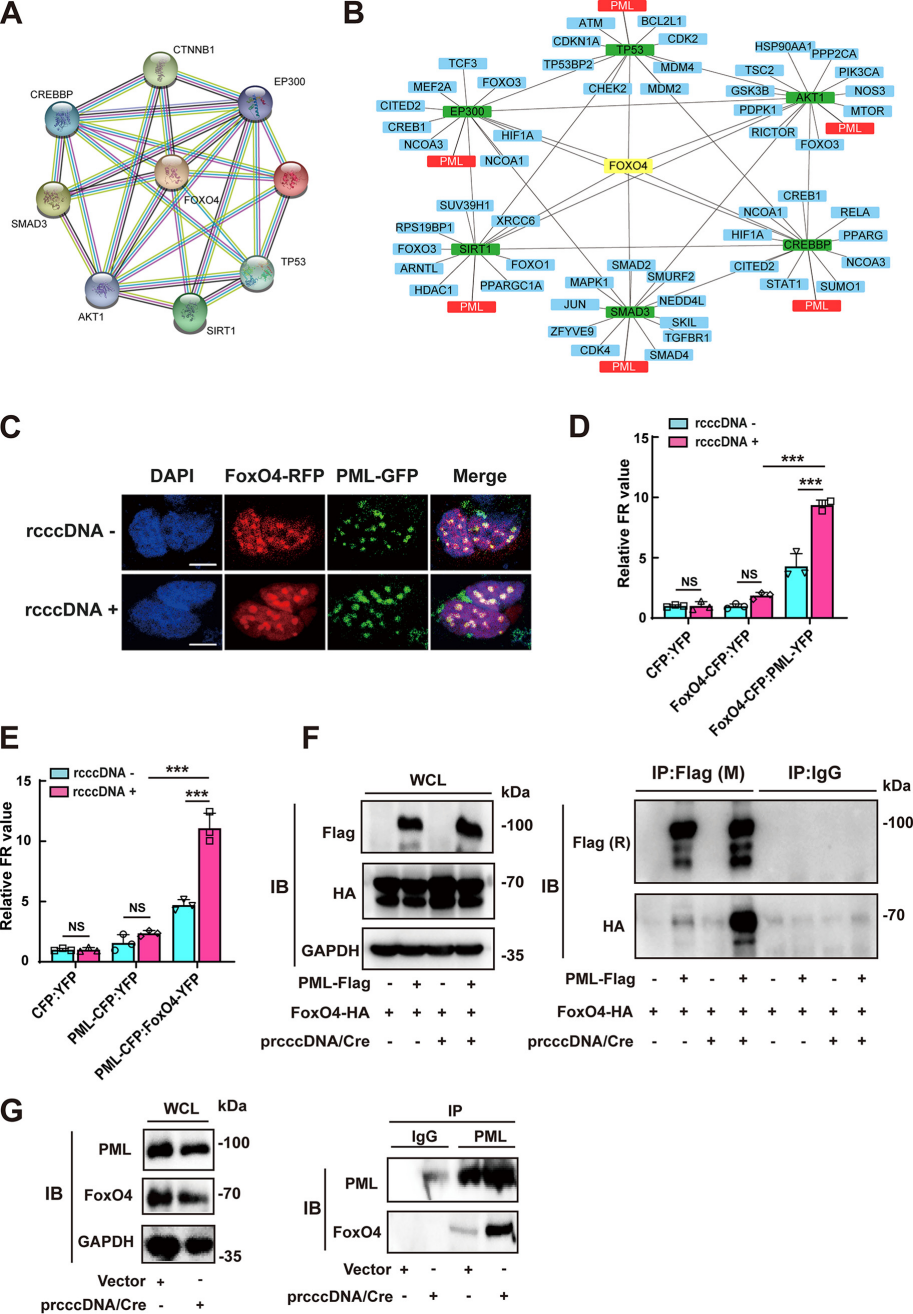
FoxO4 colocalizes with PML in PML-NB and interacts with PML. (A) FoxO4 was selected as the input in STRING database to output FoxO4-associated protein network. (B) With those FoxO4-assoicated proteins as the input, STRING networks were reconstructed and then subjected to visualization by cytoscape software. (C) FoxO4-RFP, PML-GFP, and prcccDNA/Cre were cotransfected into Huh7 cells. At 48 h posttransfection, the cells were fixed, stained with DAPI (blue) and visualized by confocal microscopy. Scale bar, 10 μm. (D and E) The association of FoxO4 and PML was determined by a FRET assay. (F) The interaction of FoxO4 and PML in the absence or presence of rcccDNA was determined by Co-IP analysis. FoxO4-HA and PML-Flag were cotransfected into Huh7 cells in the presence or absence of rcccDNA. At 48 h posttransfection, immunoprecipitation was performed with mouse M2 anti-Flag or IgG, and immunoblotting was then performed with rabbit anti-Flag, rabbit anti-HA, and GAPDH. (G) The interaction of endogenous FoxO4 and PML in the absence or presence of rcccDNA. Huh7 cells were transfected with or without prcccDNA/Cre. At 48 h posttransfection, cells were immunoprecipitated with anti-PML or control IgG and then subjected to immunoblotting with anti-FoxO4 or anti-PML. The data are shown as means ± the SD of triplicates and are representative of three independent experiments (***, *P < *0.001; NS, no significance).

To further investigate the mechanisms by which FoxO4 exerted its epigenetic suppressive effect on cccDNA, we had performed a protein-protein interaction analysis using the Search Tool for the Retrieval of Interacting Genes (STRING; https://string-db.org/) version 11.0, aiming to gather, score, and integrate all publicly available sources of protein-protein interaction information about FoxO4 (Fig. 5A). The results showed that FoxO4 might have functional associations with SMAD3, the protein kinase AKT1, the protein acetyltransferase CREBBP, EP300, and the deacetylase SIRT1, consistent with reports that FoxO family members, including FoxO4, are under the control of transforming growth factor β (TGF-β) and subjected to posttranslational modifications, such as phosphorylation and acetylation (16, 17, 27–30). STRING database analysis also showed that FoxO4 may have some functional connections with p53, which has been reported to display inhibitory effect on HBV transcription (31, 32), and data from our coimmunoprecipitation (Co-IP) assay showed that FoxO4 could indeed interact with p53 (see Fig. S4A). However, p53 appeared to be not involved in FoxO4-mediated suppression of cccDNA transcription, since there was no significant difference between HepG2 (p53 wild type), Huh7 (p53 mutation), and Hep3B (p53 deletion) in the context of FoxO4-mediated inhibition of cccDNA transcription and HBV replication (see Fig. S4B to G). Further, with each of these FoxO4-associated molecules as the input, we reconstructed STRING networks which were further visualized by using Cytoscape (version 3.8.2). Of interest, it was found that those FoxO4-associated molecules, including SMAD3, AKT1, EP300, CREBBP, SIRT1, and P53, also targeted promyelocytic leukemia protein (PML), suggesting the possible functional interactions between FoxO4 and PML (Fig. 5B). Accumulating evidence indicates that PML and PML nuclear bodies (PML-NB) play a critical role in antiviral responses and may contribute to epigenetic suppression of viruses, including HBV (9, 33, 34). We thus further determined the interaction between FoxO4 and PML in rcccDNA cell model. Data from confocal analysis revealed the colocalization between FoxO4 and PML in PML-NB (Fig. 5C), We further used fluorescence resonance energy transfer (FRET) technique to analyze the association between FoxO4 and PML by cotransfecting equal amounts of pECFP-N1-FoxO4 and pEYFP-N1-PML or equal amounts of pECFP-N1-PML and pEYFP-N1-FoxO4 into Huh7 cells in the absence or presence of prcccDNA/Cre. As shown in Fig. 5D, FoxO4 (act as the donor) produced strong FRET signal with PML (act as the acceptor), especially in the presence of rcccDNA; similar data were obtained when PML acted as the donor and FoxO4 acted as the acceptor (Fig. 5E), indicating the functional interaction between FoxO4 and PML. The Co-IP experiments also demonstrated that the exogenously expressed FoxO4-HA could interact with PML-Flag and the presence of rcccDNA appeared to enhance this interaction (Fig. 5F), and further examination of the interaction between endogenous FoxO4 and PML yielded a similar conclusion (Fig. 5G).

### PML was critical for FoxO4-mediated epigenetic suppression cccDNA.

**FIG 6:**
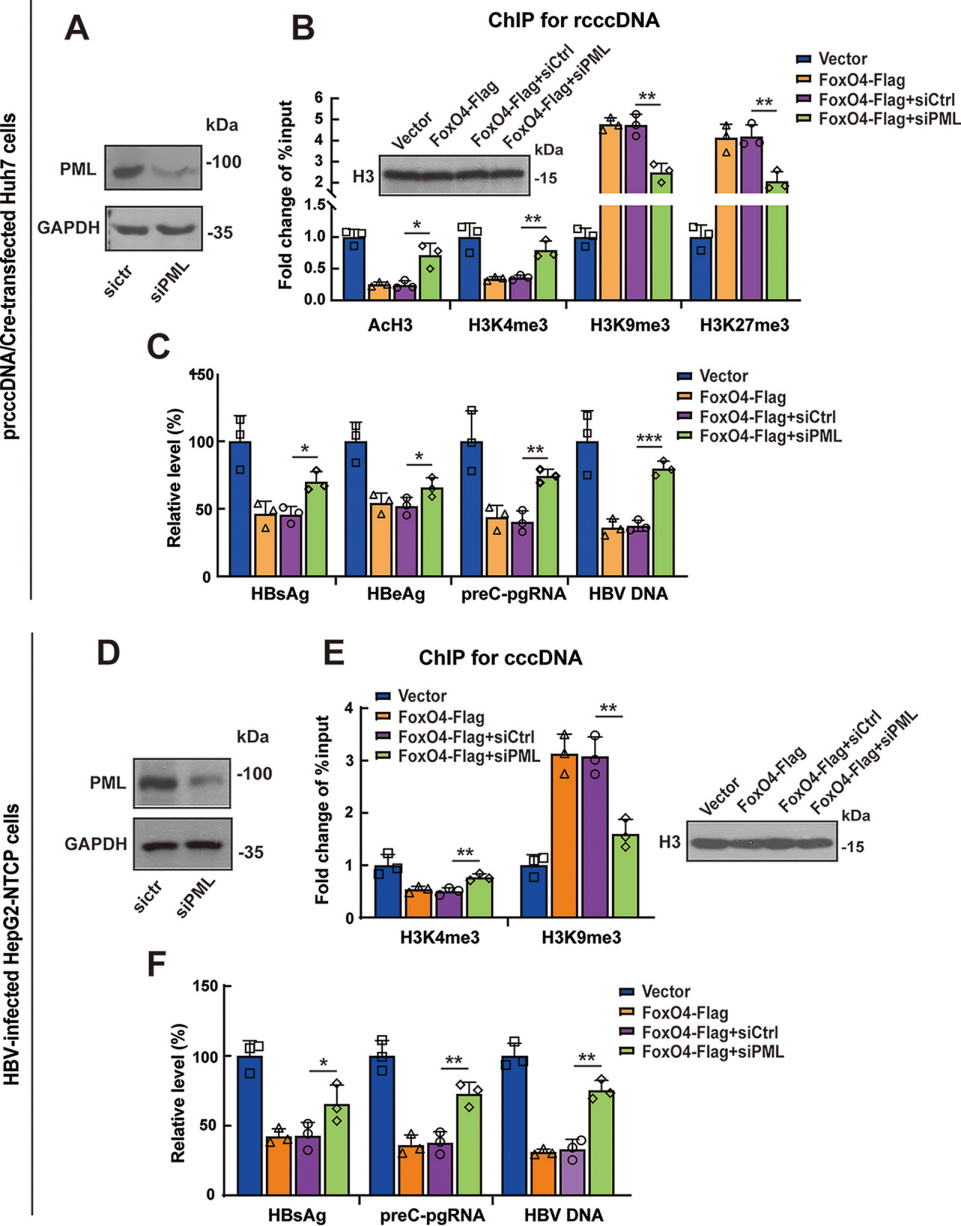
PML is involved in the FoxO4-mediated epigenetic suppression of cccDNA. (A to C) Huh7 cells were transfected with control or PML siRNA. After 48 h, the cells were further transfected with FoxO4 and prcccDNA/Cre for another 48 h. (A) The protein level of PML was determined by Western blotting. (B) The recruitment of AcH3, H3K4me3, H3K9me3, and H3K27me3 onto rcccDNA was examined by ChIP assays, and data are shown as fold change versus empty vector-transfected cells after being normalized to input and control IgG. (C) The levels of HBV proteins (HBsAg and HBeAg), preC-pgRNA, and HBV DNA were determined by ELISA, qRT-PCR, and qPCR, respectively. (D to F) HepG2-NTCP cells were electrotransfected with control or PML siRNA. After 48 h, the cells were further transfected with FoxO4-Flag, followed by infection with HBV at 10^3^ vge/cell. At day 3 postinfection, the efficacy of PML knockdown was determined by Western blotting (D), and the recruitment of H3K4me3 and H3K9me3 onto cccDNA was determined by ChIP assays as in panel B (E). (F) At day 9 postinfection, the levels of HBsAg, preC-pgRNA, and HBV-DNA were determined by ELISA, qRT-PCR, and qPCR, respectively. The data are shown as means ± the SD of triplicates and are representative of three independent experiments (*, *P < *0.05; **, *P < *0.01; ***, *P < *0.001).

Since the data described above demonstrated that FoxO4 could colocalize with PML in PML-NB and interact with PML in HBV replicating cells, we further determined the role of PML in FoxO4-mediated epigenetic suppression of HBV cccDNA. Results from a ChIP assay showed that PML downregulation by siRNA technique significantly reversed FoxO4-mediated epigenetic suppression of rcccDNA, as evidenced by the increased binding of euchromatin markers (AcH3 and H3K4me3) and the decreased binding of heterochromatin markers (H3K9me3 and H3K27me3) to rcccDNA (Fig. 6A and B). Accordingly, knockdown of PML significantly attenuated FoxO4-mediated inhibition of rcccDNA-driven transcription and HBV replication (Fig. 6C). We had further investigated the role of PML in FoxO4-mediated epigenetic suppression of cccDNA in HBV-infected HepG2-NTCP cells. Consistent with the data obtained from rcccDNA cell model, knockdown of PML significantly reversed FoxO4-mediated epigenetic suppression of cccDNA (Fig. 6D and E), and the following inhibition of cccDNA-driven transcription and HBV replication (Fig. 6F). Since our previous investigation showed that HNF4α was involved in FoxO4-mediated inhibition of cccDNA transcription, we tested the effect of PML knockdown on the expression level of HNF4α. We found that PML downregulation did not affect the expression level of HNF4α in the absence or presence of FoxO4 (see Fig. S5), indicating that FoxO4 exerted the epigenetic suppression of cccDNA independent of its inhibition of HNF4α. Taken together, our data demonstrated that PML played a critical role in FoxO4-mediated epigenetic suppression of HBV cccDNA, which was independent of its downregulation of HNF4α.

### FoxO4 is downregulated in HBV-infected hepatocytes and human liver tissues and is negatively correlated with cccDNA transcriptional activity.

**FIG 7:**
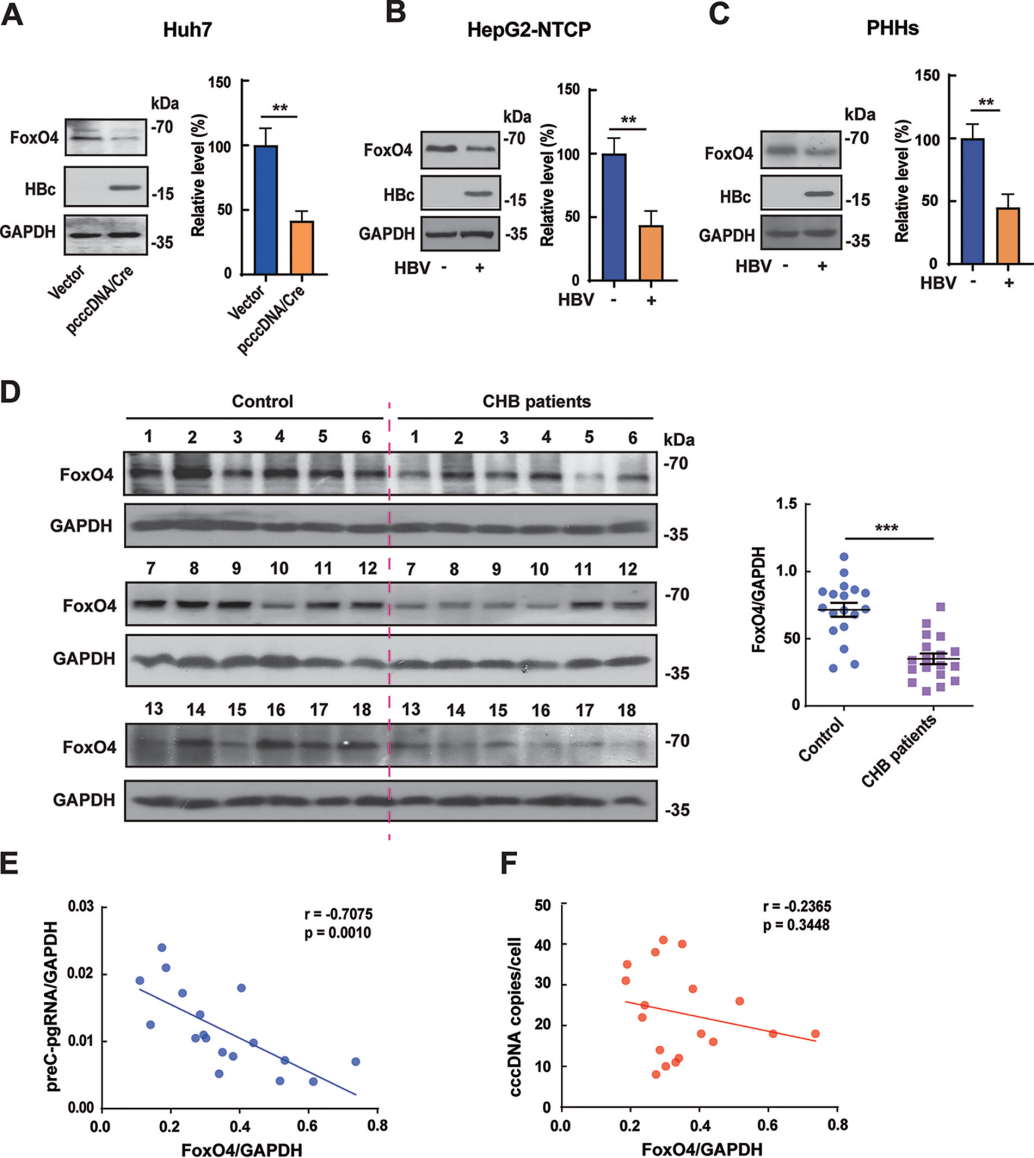
The expression of FoxO4 is negatively correlated with HBV cccDNA in HBV-infected hepatocytes and human liver tissue. (A) Huh7 cells were transfected with control empty vector or prcccDNA/Cre. At 48 h posttransfection, the level of FoxO4 protein was determined by Western blotting. (B) HepG2-NTCP cells were infected with HBV at 10^3^ vge/cell for 9 days, and the level of FoxO4 protein was determined as in panel A. (C) Primary hepatocytes (PHHs) were infected with HBV at 10^2^ vge/cell for 9 days, and the level of FoxO4 protein was determined as in panel A. (D) The protein level of FoxO4 in liver tissues from control individuals and CHB patients (*n* =18) was determined by Western blotting. (E and F) Correlations between FoxO4 protein and HBV preC-pgRNA (E) or HBV cccDNA (F) in CHB patients (*n* = 18). The data are shown as means ± the SD of triplicates and are representative of three independent experiments (**, *P < *0.01; ***, *P < *0.001).

Accumulating evidence indicates that the interplay between host and HBV plays a crucial role in determining the fate of infection (35, 36). In our previous investigation (19), we found that FoxO4 was downregulated at the protein level but not at the mRNA level in HBV replication-competent plasmid (pHBV1.3)-transfected Huh7 cells and HepG2 cells. In the present investigation, we further tested the effect of HBV on FoxO4 expression in the cccDNA system. Our data showed that HBV could significantly downregulate the protein level of FoxO4 in prcccDNA/Cre-transfected Huh7 cells (Fig. 7A) and in HBV-infected HepG2-NTCP cells (Fig. 7B), as well as in HBV-infected primary human hepatocytes (PHHs) (Fig. 7C). Furthermore, we detected the expression levels of FoxO4 in human liver biopsy specimens and found that the levels of FoxO4 protein were significantly downregulated in HBV-positive CHB patients (Fig. 7D). Of note, we found that there existed a significant negative correlation between the levels of FoxO4 protein and HBV preC-pgRNA (Fig. 7E), but not between FoxO4 protein and HBV cccDNA itself (Fig. 7F) in CHB patients, further suggesting the suppressive effect of FoxO4 on cccDNA function under physiological conditions.

## DISCUSSION

Nuclear-located cccDNA plays a central role in HBV transcription and replication and is considered to be a primary mechanism for HBV persistence. Targeting cccDNA is therefore a promising therapeutic strategy against chronic hepatitis B.

In the present investigation, our data showed that FoxO4 significantly suppressed cccDNA-mediated transcription and HBV replication both *in vitro* and *in vivo*, while it had little effect on the level of cccDNA itself. A growing number of studies demonstrate that epigenetic modification may play a crucial role in cccDNA transcription and HBV replication. FoxO proteins, sharing a DNA-binding Forkhead box domain, mainly function as transcription factors in the nucleus and may act as epigenetic effectors (23, 24). We thus wondered whether FoxO4 could bind to cccDNA and lead to the epigenetic silencing of cccDNA. Our data showed that FoxO4 could indeed bind to cccDNA and lead to its heterochromatinization efficiently, as demonstrated by the decrease of active chromatin marks AcH3 and H3K4me3 and the increase of repressive chromatin marks H3K9me3 and H3K4me3 on HBV cccDNA, indicating that FoxO4 might cause the epigenetic suppression via enhancing the heterochromatinization of cccDNA. Our previous investigation revealed that the innate DNA sensor IFI16 could inhibit the function of HBV cccDNA by integrating innate immune activation and epigenetic suppression (8), we had therefore been interested in the possible role of IFI16 in FoxO4-mediated antiviral activities. However, our data revealed that FoxO4 failed to activate innate immune signaling in a cccDNA cell model and IFI16 knockdown also did not have a significant effect on FoxO4-mediated inhibition of HBV, indicating that IFI16 might not contribute to the anti-cccDNA activity of FoxO4.

Our previous data showed that HNF4α was implicated in FoxO4-mediated inhibition of HBV transcription via suppressing HBV core promoter (19). It is reported that HNF4α may display epigenetic activities in some circumstances. For example, HNF4α was involved in the regulation of *de novo* DNA methylation by DNMT3s in epithelial-to-mesenchymal transition (25), and HNF4α could also trigger epigenetic silence of the human PED gene by inducing acetylation or methylation of promoter-bound histones (26). We thus explored whether HNF4α was involved in FoxO4-mediated inhibition of cccDNA epigenetic modification. Our data showed that HNF4α expression significantly reversed FoxO4-mediated inhibition of HBV core promoter activity, consistent with our previous investigation (19). However, it failed to affect FoxO4-mediated epigenetic suppression of cccDNA, indicating that HNF4α might not participate in FoxO4-mediated epigenetic suppression of HBV cccDNA.

To further investigate the mechanisms by which FoxO4 exerted its epigenetic suppressive effect on cccDNA, we performed STRING database analysis for FoxO4. The results showed that FoxO4 displayed functional association with SMAD3, AKT1, EP300, CREBBP, and SIRT1, in agreement with reports that FoxO4 is under the control of TGF-β and subjected to posttranslational modification, including phosphorylation and acetylation (16, 17, 27–30). The STRING assay also showed the possible association of FoxO4 with P53, and our Co-IP assays indeed revealed the interaction between them, consistent with previous investigation (37, 38). However, p53 appeared to be not involved in FoxO4-mediated inhibition of HBV cccDNA, since there were no significant differences in FoxO4 inhibition of cccDNA transcription and HBV replication between cells with p53 wild type and those with p53 mutation or deletion. Further, STRING networks were reconstructed with each of FoxO4-related proteins as the input and then subjected to analysis by Cytoscape software. Of interest, it was found that those FoxO4-related agents appeared to also target PML. It is now evident that PML plays an essential role in proper formation and the integrity of nuclear bodies (NBs), which have been implicated in a wide variety of biological processes, such as senescence, stemness, and antiviral defense (39). Accumulating reports indicate that PML-NBs might cause the epigenetic silencing of viral genomes, thus interrupting the life cycles of several viruses (9, 40, 41). Of interest, Zhou et al. (42) also reported that both FoxO4 and PML were under the regulation of UHRF1, an epigenetic regulator, suggesting their possible coordination in epigenetic processes. We thus further determined the interaction between FoxO4 and PML. Cofocal experiments indicated the colocalization between FoxO4 and PML in nuclear bodies. Further FRET analysis and Co-IP experiments analysis confirmed the interaction between FoxO4 and PML, especially in the presence of rcccDNA. In agreement with our data, Baar et al. (37) also demonstrated that FoxO4 resides within PML bodies by using structured illumination microscopy.

To investigate the role of PML in FoxO4-mediated epigenetic regulation of cccDNA, we downregulated the expression of PML by siRNA technique and then tested the effect of FoxO4 on cccDNA. It was found that knockdown of PML indeed significantly decreased the FoxO4-mediated epigenetic suppression of cccDNA and subsequently attenuated the FoxO4-mediated inhibition of cccDNA transcription and HBV replication. We had also tested the effect of PML knockdown on HNF4α expression and found that PML downregulation did not affect the expression of HNF4α in the absence or presence of FoxO4, further indicating that FoxO4 exerted its epigenetic suppression of cccDNA independent of its inhibitory effect on HNF4α expression. It was reported that HBV x protein (HBx) is critical for cccDNA transcription, and the transcriptional repression of HBV with HBx mutation (HBVX–) correlates not only with a decrease of active histone markers (H3K4me3) but also with the deposition of repressive markers (H3K9me3 and H3K27me3) on cccDNA (43). Since several lines of evidence indicate that the chromosome 5/6 complex (Smc5/6) could block cccDNA transcription through degradation of HBx (44, 45) and the Smc5/6 complex has been reported to localize to PML bodies (34), we therefore examined the effect of FoxO4 on the Smc5/6 complex. Our data showed that FoxO4 did not affect the expression of SMC5 and SMC6; accordingly, it was found that FoxO4 exerted its anti-HBV activity independent of HBx (see Fig. S6). In addition, we had tested the effect of FoxO4 on PML expression; however, we found that FoxO4 expression failed to affect the expression level of PML significantly (data not shown). Whether FoxO4 affects the posttranslation modification of PML or whether other proteins (e.g., some PML-ND components) were required for forming a multiunit protein complex with FoxO4 and PML and thus contributed to FoxO4-mediated epigenetic suppression of HBV cccDNA merits further investigation.

Accumulating evidence demonstrates that there may exist interplays between host and HBV, and HBV has developed strategies against the host defense for the establishment of persistent viral infection (35). Our data showed that in HBV cccDNA cell models, including prcccDNA/Cre-transfected Huh7 cells, HBV-infected HepG2-NTCP cells and PHHs, HBV could significantly inhibit the expression of FoxO4 protein. Further, it was found that the expression level of FoxO4 was significantly downregulated in liver tissue of HBV-positive CHB patients. Of note, the level of FoxO4 protein was negatively correlated with HBV transcripts, but not with the cccDNA level itself in liver tissues of CHB patients, further suggesting that FoxO4 might act as a suppressor of cccDNA transcription under physiological conditions.

In summary, our data demonstrated that FoxO4 could efficiently suppress cccDNA-mediated transcription and HBV replication without affecting the level of cccDNA itself. Further mechanistic study showed that, besides its inhibition of HBV core promoter via downregulating HNF4α, FoxO4 could bind to cccDNA and lead to the heterochromatinization of cccDNA through interaction with PML (see Fig. S7). Of note, we found that FoxO4 protein was downregulated in HBV-infected hepatocytes and human liver tissue and was negatively correlated with cccDNA transcriptional activity in CHB patients, indicating that HBV might dampen the expression of FoxO4 for its persistent infection.

## MATERIALS AND METHODS

### Cell culture and patient specimens.

Huh7, HepG2, and Hep3B cells were cultured in Dulbecco modified Eagle medium supplemented with 10% fetal bovine serum, 100 μg/mL penicillin, and 100 μg/mL streptomycin. For the culture of HepG2 cells stably expressing sodium taurocholate cotransporting polypeptide (HepG2-NTCP), the culture media were supplemented with puromycin at the concentration of 4 μg/mL. PHHs were isolated from surgical removed liver tissues as previously described (46). All cells were maintained in an incubator containing 5% CO_2_ at 37°C. Human liver tissue samples were obtained from Zhongshan Hospital (Fudan University, Shanghai, China). For the control group, liver tissues were collected from patients who underwent surgical resection for benign hepatic lesions. Patients with the following criteria were excluded: already under antiviral therapy, infection with HCV, HDV, and HIV decompensated liver disease, steatohepatitis or autoimmune liver diseases. The characteristics of CHB patients and control individuals in this study are shown in Table S1 in the supplemental material. This study was conducted in accordance with the Declaration of Helsinki. Approval for the use of human subjects was obtained from the research Ethics Committee of Fudan University, and informed consent was obtained from each individual enrolled in this study.

### Plasmids or siRNA transfection and HBV infection.

Huh7 cells were cotransfected with prcccDNA plus pCMV-Cre (prcccDNA/Cre) to produce recombinant cccDNA (rcccDNA) as reported by Qi et al. (21). The high levels of rcccDNA produced in this model had been confirmed to be a surrogate for the natural HBV cccDNA minichromosome. Linear HBV monomers were released from pHBV-SapI with restriction endonuclease SapI as described previously (47). FoxO4-Flag (catalog no. 17549) and PML-IV-Flag (catalog no. 62804) were obtained from Addgene. The FoxO4 or PML-IV gene was further subcloned into pEGFP-N1, pERFP-N1, pECFP-N1, and pEYFP-N1, respectively. To investigate the effect of FoxO4 overexpression on cccDNA in Huh7 cells, prcccDNA/Cre or linear HBV monomers were transfected into Huh7 cells, together with FoxO4-Flag or control empty vector, using Lipofectamine 2000 reagent (Invitrogen, Carlsbad, CA) according to the manufacturer’s instructions. For the siRNA experiments, siRNAs specific for FoxO4 or PML (see Table S1) were transfected into Huh7 cells by using siRNA-Mate transfection reagent (GenePharma, Shanghai, China). HepG2-NTCP cells were electrotransfected with plasmids or siRNAs using Amaxa Nucleofector (Amaxa GmbH, Cologne, Germany) as described previously (8, 19). In all transfection assays, pCMV-β-gal was cotransfected to normalize the transfection efficiency. For HBV infection experiments, HepG2-NTCP cells were infected with HBV at 10^3^ virus genome equivalents (vge)/cell in the presence of 4% PEG-8000. At ~16 h after infection, the cells were washed three times with phosphate-buffered saline (PBS), and then maintained in medium supplemented with 2.5% dimethyl sulfoxide. To infect PHHs, cells were incubated with HBV at 10^2^ vge/cell as described previously (8, 48, 49).

### Hydrodynamics-based transfection in mice.

Four-week-old C57BL/6 male mice were hydrodynamically injected with 4 μg of precursor plasmid cccDNA (prcccDNA), 4 μg of Cre-recombinase expression plasmid (pCMV-Cre), and 4 μg of FLAG-tagged plasmid expressing mouse FoxO4 (mFoxO4-Flag) or vector in a volume of PBS equivalent to 8% of the mouse body weight through the tail veins within 5 to 8 s, as described previously. At 4 days after the injection, mouse liver tissues and sera were collected for further analysis. Animal experiments were performed in accordance with the *Guide for the Care and Use of Medical Laboratory Animals* (Ministry of Health, China) and approved by the Animal Ethics Committee of Fudan University (Shanghai, People’s Republic of China).

### Western blotting.

As described as previously (8, 19, 48, 49), cells or liver tissues were lysed at 4°C in lysis buffer (25 mM Tris-HCl [pH 7.6], 150 mM NaCl, 1% NP-40, and 0.1% sodium dodecyl sulfate [SDS] plus protease inhibitor cocktail and phenylmethylsulfonyl fluoride). Extracted proteins were separated by SDS-PAGE and transferred onto polyvinylidene fluoride membranes. The membranes were then blocked with 5% nonfat milk for 1 h at room temperature and incubated overnight at 4°C with primary antibodies. The following day, incubation with peroxidase-conjugated secondary antibodies was carried out for another 45 min. Immunoblot signals were examined using SuperSignal West Femto maximum sensitivity substrate (Thermo scientific). The antibodies used in this study are available in Table S2 in the supplemental material.

### HBV protein analysis.

At indicated days after HBV transfection or infection, the levels of hepatitis B surface antigen (HBsAg) and hepatitis B e antigen (HBeAg) in cell culture supernatants or mouse sera were measured by ELISA (Kehua Biotech, Shanghai, China).

### qRT-PCR.

As described previously (8, 19, 48, 49), total RNA from cells or liver tissues was extracted using TRIzol (TaKaRa, Dalian, China) according to the manufacturer’s protocol and reverse transcribed into cDNA, followed by quantitative real-time PCR using a Roche LightCycler 480 II and Hieff qPCR SYBR Green Master Mix (Yeasen Biotechnology, Shanghai, China). The primers used in this study are provided in Table S3. To determine HBV RNAs and preC-pgRNA, a PrimeScript RT reagent kit with genomic DNA (gDNA) Eraser (TaKaRa) was used, in which gDNA Eraser removed the contaminating transfected HBV DNA.

### Intracellular core particle-associated HBV DNA extraction and analysis.

Extraction and quantification of core particle-associated HBV DNA were performed as described previously (8, 48–51). For Southern blot analysis, HBV DNA was separated on a 1% agarose gel, transferred onto a positively charged nylon membrane (Roche, Mannheim, Germany), and hybridized with a digoxigenin-labeled full-length HBV probe.

### Extraction and quantification of HBV cccDNA.

The cccDNA was isolated using a modified Hirt’s extraction procedure as described previously (8, 49, 52). Briefly, the pelleted cells were lysed in lysis buffer (50 mM Tris-HCl [pH 8.0], 1 mM EDTA, 0.2% NP-40, and 150 mM NaCl), followed by centrifugation at 12,000 × *g* for 30 min at 4°C. Then, the pellets were dissolved in nuclear lysis buffer (6% SDS, 0.1 M NaCl) and neutralized with 3 M potassium acetate (pH 4.8). The protein-free DNA was further extracted with phenol and chloroform and then precipitated with ethanol. Extracted DNA was further treated for 1 h at 37°C with Plasmid-Safe DNase (Epicentre Biotechnologies, Madison, WI) and then subjected to qPCR with primers for cccDNA (see Table S3). The absence of contaminating genomic DNA was further confirmed by the negative PCR results for β-globin.

### Chromatin immunoprecipitation assay.

A chromatin immunoprecipitation (ChIP) assay was performed as described previously (8, 19, 22, 53). Briefly, Huh7 cells or HepG2-NTCP cells were treated with 1% formaldehyde for 10 min to generate DNA-protein cross-links. Liver tissues were homogenized in lysis buffer (5 mM PIPES, 85 mM KCl, 0.5% NP-40) and incubated for another 10 min at 4°C. After centrifugation, the pelleted nuclei were fixed in 1% formaldehyde for 30 min at 4°C. Sonication was performed to generate chromatin fragments of 200 to 300 bp, which were subsequently immunoprecipitated with control IgG and antibodies as shown in Table S2. After the reverse cross-linking, DNA was extracted and further subjected to the treatment by Plasmid-Safe ATP-Dependent DNase (Epicentre Biotechnologies, Madison, WI) for 1 h. The rcccDNA or cccDNA was then finally analyzed by real-time qPCR with corresponding primers as shown in Table S3. The data are presented as fold differences relative to input and values obtained by control IgG using the following formula:

$$2^{\left[(C_{T}\text{IgG}–C_{T}\text{Input})-(C_{T}\text{Ab}–C_{T}\text{Input})\right]}$$

where *C_T_* is the threshold cycle, IgG is the control IgG, Ab is the specific antibody, and Input is the starting chromatin material from each experiment.

### Luciferase reporter assay.

The activity of HBV core promoter was examined as described previously (19, 50). Briefly, HBV core promoter-dependent luciferase reporter plasmid CpLUC and FoxO4-Flag were transfected into Huh7 or HepG2-NTCP cells in the absence or presence of HNF4α. At 48 h posttransfection, the luciferase activity in the lysates of transfected cells was determined by using a luciferase reporter assay system (Promega, Madison, WI).

### Coimmunoprecipitation assay.

Huh7 cells transfected with indicated plasmids were collected, washed with PBS, lysed in lysis buffer (1% NP-40, 0.5% sodium deoxycholate, 150 mM NaCl, 20 mM Tris-HCl [pH 7.4]) with protease inhibitor cocktail, and centrifuged at 12,000 × *g* for 10 min at 4°C, and the supernatant was then collected. For each sample, the lysate was precleared with protein A-agarose beads at 4°C for 1 h, and then the supernatant and anti-Flag M2 agarose bead slurry (Sigma-Aldrich, St. Louis, MO) or protein A-agarose beads preincubated with control IgG were incubated at 4°C overnight on a rotator. The following day, beads were washed four times with PBST (PBS, 1% Triton X-100), boiled in sample-loading buffer for 3 to 5 min, and subjected to Western blotting.

### Fluorescence imaging.

Huh7 cells were seeded on round glass coverslips and transfected with corresponding fluorescence plasmids. At 48 h posttransfection, the cells were fixed at room temperature for 30 min with 4% paraformaldehyde. Subsequently, the cells were washed with PBS for three times, and DAPI was added for 3 to 5 min to stain nuclei. Fluorescence imaging was performed using a Leica TCS SP8 confocal microscope. Images of each sample were acquired using identical microscope, laser, filter, and camera settings, and ImageJ software (version 1.8.0) was used for image analysis in all cases.

### Fluorescence resonance energy transfer.

Huh7 cells were seeded in 96-well plates and transfected with equal amounts of pECFP-N1-FoxO4 and pEYFP-N1-PML or equal amounts of pECFP-N1-PML and pEYFP-N1-FoxO4, together with or without precursor plasmid cccDNA (prcccDNA) plus Cre recombinase expression plasmid (pCMV-Cre). At 48 h after transfection, cells were washed with PBS for three times and then subjected to fluorescence intensity detection by using a Tecan microplate reader (Tecan, Mannedorf, Switzerland). The “fluorescence bottom reading” module was applied, and the parameters set for measurement, including wavelength and width, were as follows: CFP filter channel (excitation, 433/20 nm; emission, 478/20 nm), YFP filter channel (excitation, 513/20 nm; emission, 558/20 nm), and FRET filter channel (excitation, 433/20 nm; emission, 558/20 nm). For the statistical chart, the FRET signal was quantified by the FRET ratio (FR), which was calculated using the formula: FR = [FRET – (*a* × CFP)]/(*b* × YFP), in which CFP, YFP, and FRET correspond to the fluorescence intensity values acquired from the above three measurements, *a* represents the ratio of FRET channel intensity to CFP channel intensity for pure CFP protein, and *b* represents the ratio of FRET channel intensity to YFP channel intensity for pure YFP protein, as described previously (54–56).

### Statistics.

Data were expressed as means ± the standard deviations (SD). Statistical significance was tested using a two-tailed, unpaired Student *t* test (for two sample comparisons) or one-way analysis of variance (ANOVA) with Dunnett’s multiple comparison correction data (for multiple comparisons). Pearson’s correlation was used for correlation analysis. Statistical analysis was performed using the GraphPad Prism software (version 9.1.2). *P *values of *<*0.05 were considered statistically significant.
