# Supplementary material for: Forkhead O Transcription Factor 4 Restricts HBV Covalently Closed Circular DNA Transcription and HBV Replication through Genetic Downregulation of Hepatocyte Nuclear Factor 4 Alpha and Epigenetic Suppression of Covalently Closed Circular DNA via Interacting with Promyelocytic Leukemia Protein
Source: J Virol. 2022 Jun 13;96(13):e00546-22. doi: 10.1128/jvi.00546-22 (PMC9278149; doi:10.1128/jvi.00546-22)
Supplement: Supplemental file 1 — Fig. S1 to S7, Tables S1 to S3. Download jvi.00546-22-s0001.pdf, PDF file, 0.7 MB [file jvi.00546-22-s0001.pdf]

Forkhead O transcription factor 4 restricts HBV covalently closed circular DNA transcription and HBV replication through genetic downregulation of hepatocyte nuclear factor 4 alpha and epigenetic suppression of covalently closed circular DNA via interacting with promyelocytic leukemia protein

Yuqi Li[1#], Minjing He[1#], Ruijie Gong[1#], Ziteng Wang[1], Lin Lu[1], Shu Peng[1], Zhiyun Duan[1], Ying Feng[1], Yi Liu[3*] and Bo Gao[1,2*]

[#]These authors contribute equally to this work.

[1]From the Department of Immunology, School of Basic Medical Sciences, Shanghai Medical College of Fudan University, Shanghai 200032, P.R. China

[2]Liver Cancer Institute, Zhongshan Hospital, Fudan University, Shanghai 200032, P.R. China

[3]Department of Digestive Diseases, Huashan Hospital, Fudan University, Shanghai 200040, P.R. China

*Corresponding authors: Bo Gao, E-mail: gaobofd@126.com; or Yi Liu, E-mail: hsliuyi0205@163.com

Supplemental Figures and Tables

Figure S1

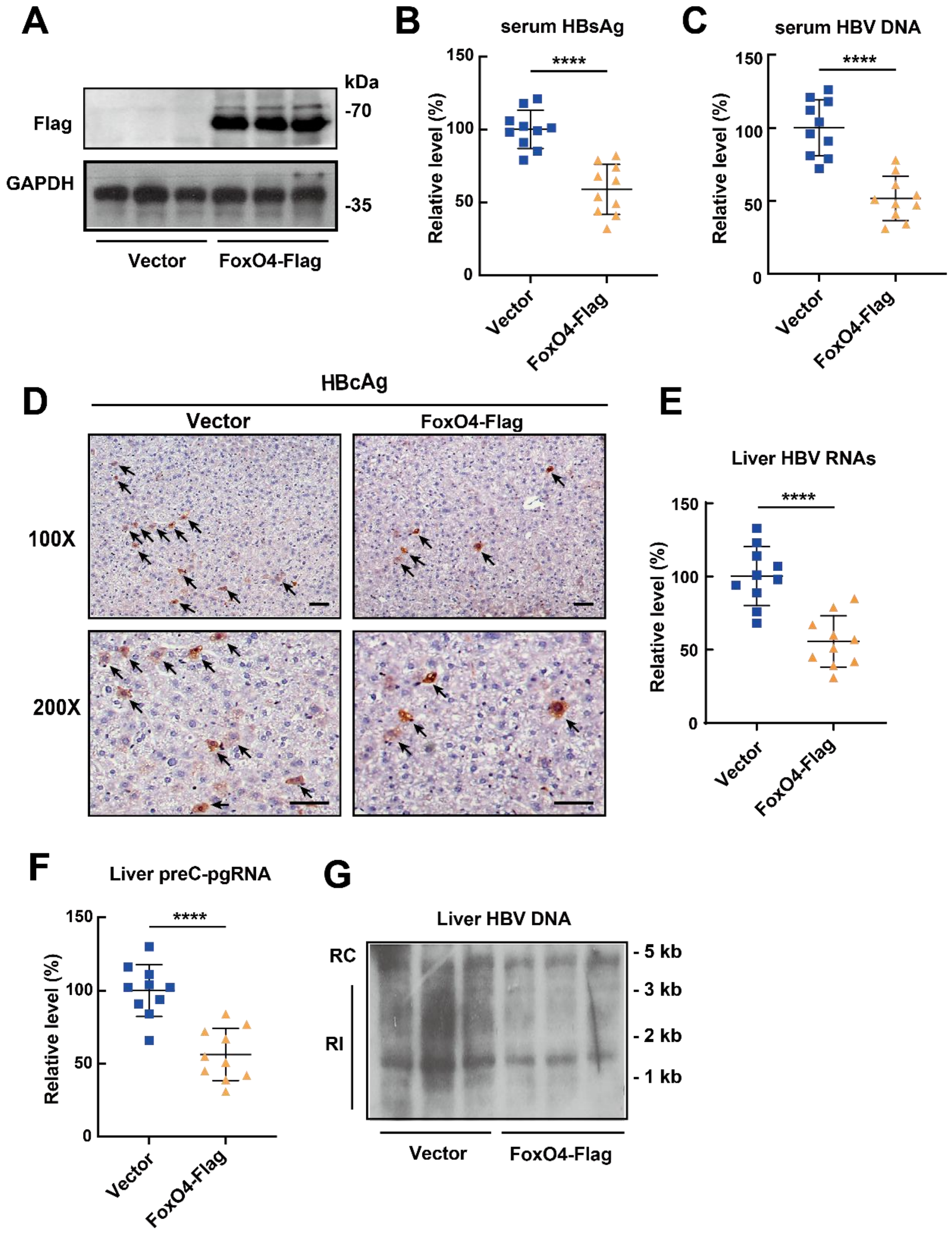

Figure S1. FoxO4 inhibits rcccDNA-mediated transcription and HBV replication in mice. Mice were injected intravenously with prcccDNA/Cre and FoxO4-Flag or vector using HGT technique (n = 5). 4 days post-injection, mice liver tissues and sera

were collected. **(A)** The protein level of FoxO4-Flag in mice liver tissues was determined by Western blotting using antibodies against Flag and GAPDH. **(B, C)** The levels of HBsAg and HBV DNA in mice sera were determined by ELISA and qPCR, respectively. **(D)** The level of HBcAg in the liver tissues of mice was analyzed by immunohistochemical staining. **(E, F)** The levels of HBV RNAs and preC-pgRNA in the liver tissues of mice were analyzed by qRT-PCR. **(G)** The level of HBV DNA in the liver tissues of mice was determined by Southern blotting. Data are shown as means ± SD and are representative of three independent experiments. Scale bar: 100 µm. ****$P < 0.0001$. NS, no significance; RI, replicative intermediates.

Supporting FIG. S2

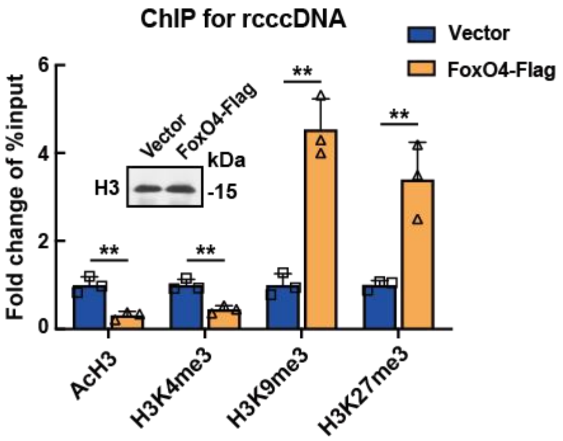

Supporting FIG. S2. FoxO4 displays epigenetic suppressive activity on rcccDNA *in vivo.* Mice were injected with prcccDNA/Cre and FoxO4-Flag or vector using HGT technique ($n = 5$). 4 days post-injection, mice liver tissues were collected. The effect of FoxO4 on the recruitment of AcH3, H3K4me3, H3K9me3, and H3K27me3 onto rcccDNA in mice liver tissues was determined by ChIP assays. Data are shown as fold change to control empty vector-transfected cells after normalized to input and control IgG. The value obtained from control siRNA-transfected and IRF-1-untreated mice was set to 1. Data are shown as means ± SD and are representative of three independent experiments. **$P < 0.01$.

Supporting FIG. S3

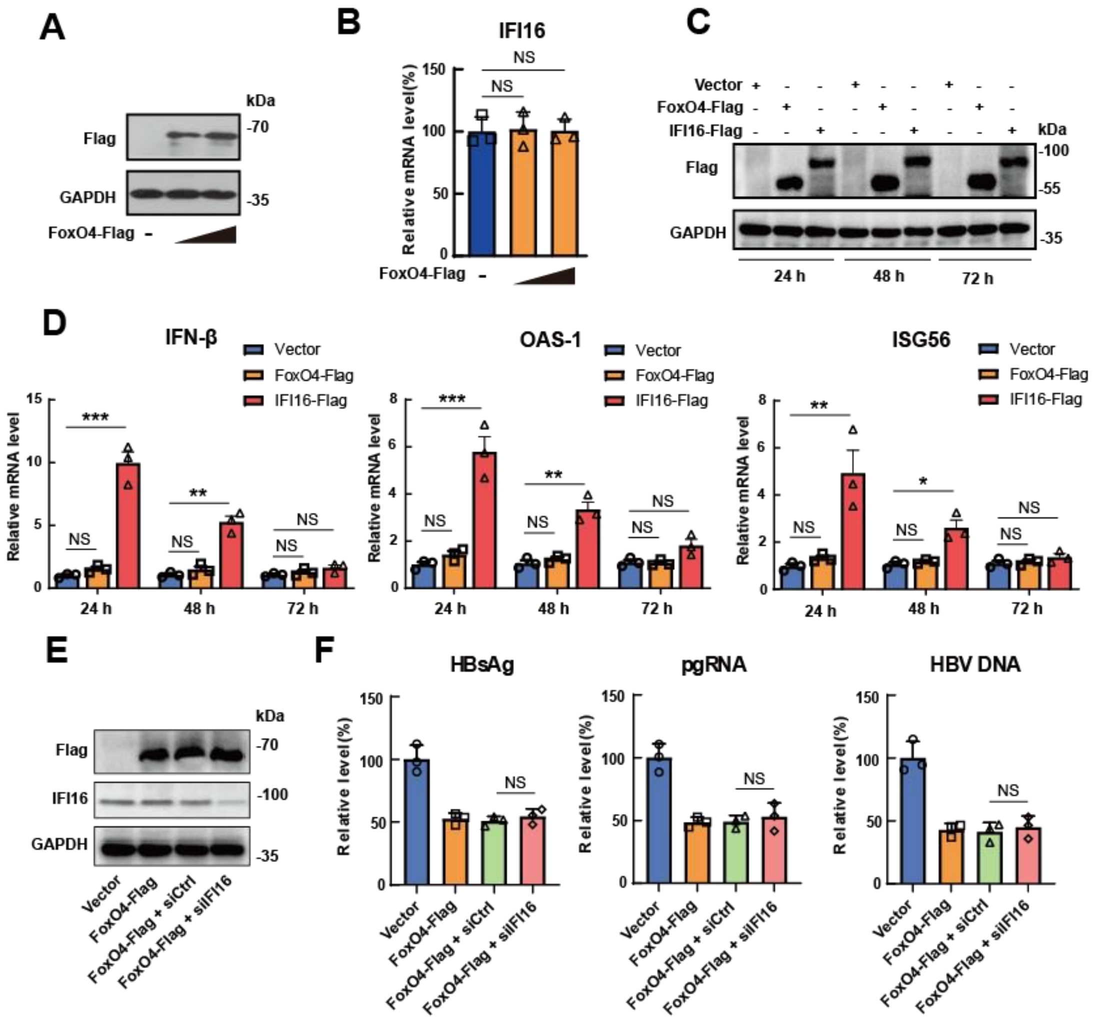

Supporting FIG. S3. The role of IFI16 in FoxO4-mediated anti-cccDNA activity. **A, B:** FoxO4-Flag was transfected into Huh7 cells for 48 h, and the protein level of exogenous FoxO4-Flag was determined by Western blotting (A), and the mRNA level of IFI16 was determined by qRT-PCR (B). **C, D:** Empty control vector, FoxO4-Flag or IFI16-Flag was transfected into Huh7 cells together with prcccDNA/Cre for different time points (24, 48, 72 hours). The exogenously-expressed FoxO4-Flag and IFI16-Flag were detected by Western blotting (C), and the mRNA levels of IFN-β, OAS1 and ISG56 (D) were examined by qRT-PCR for the indicated time points. **E, F**: Huh7 cells were transfected with IFI16 siRNA for 48 h, and the cells were further transfected with FoxO4-Flag for another 48 h. The expression level of exogenous FoxO4 was determined by Western blotting (E), and the HBV proteins, HBV transcripts and HBV DNA was examined by ELISA, qRT-PCR and qPCR,

respectively (F). Data are shown as means ± SD and are representative of three independent experiments. *$P < 0.05$; **$P < 0.01$; ***$P < 0.001$. NS, no significance.

Supporting FIG. S4

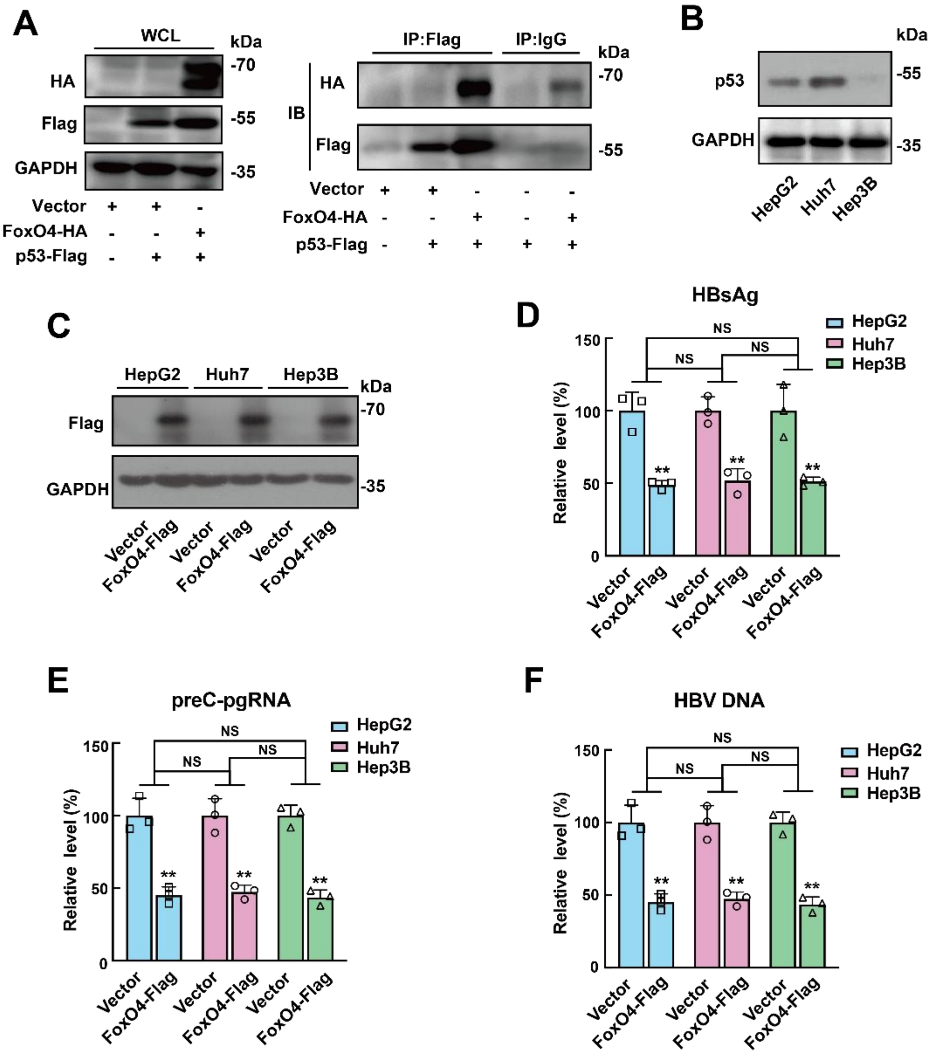

Supporting FIG. S4. p53 appears to be not involved in FoxO4-mediated transcriptional repression of rcccDNA. (A) FoxO4-HA and p53-Flag were co-transfected into Huh7 cells. After 48 h transfection, immunoprecipitation was performed using mouse M2 anti-Flag or IgG, and immunoblotting was then performed using rabbit anti-Flag, rabbit anti-HA and GAPDH. (B) The protein level of p53 in HepG2, Huh7 and Hep3B cells was determined by Western blotting using antibodies against p53 and GAPDH. (C-F) The role of p53 in FoxO4-medaited suppression of rcccDNA-driven transcription and HBV replication in HepG2, Huh7 and Hep3B cells was determined by ELISA, qRT-PCR and qPCR, respectively. Data

are shown as means ± SD and are representative of three independent experiments. $^{**}P < 0.01$. NS, no significance. The differences within and between groups were compared by Student's t-test and two-way analysis of variance (ANOVA), respectively.

Supporting FIG. S5

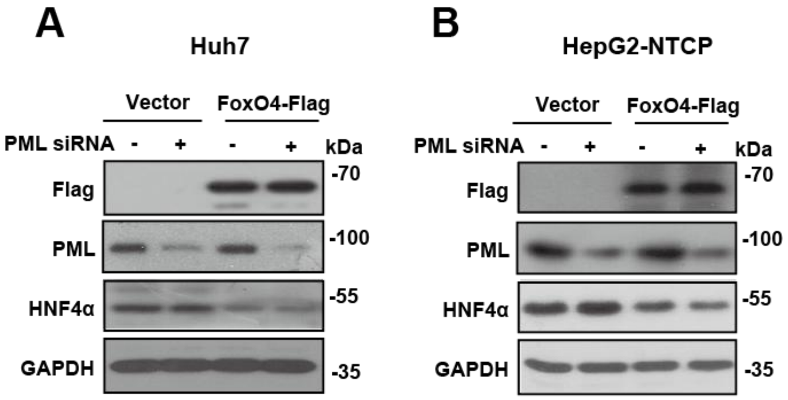

Supporting FIG. S5. PML do not affect the expression of HNF4α in the absence or presence of FoxO4. Huh7 cells (A) or HepG2-NTCP cells (B) were transfected with control or PML siRNA. 48 hours later, cells were further transfected with FoxO4-Flag or control empty vector for another 48 hours. The expression levels of FoxO4-Flag, PML and HNF4α were determined by Western blotting.

Supporting FIG. S6.

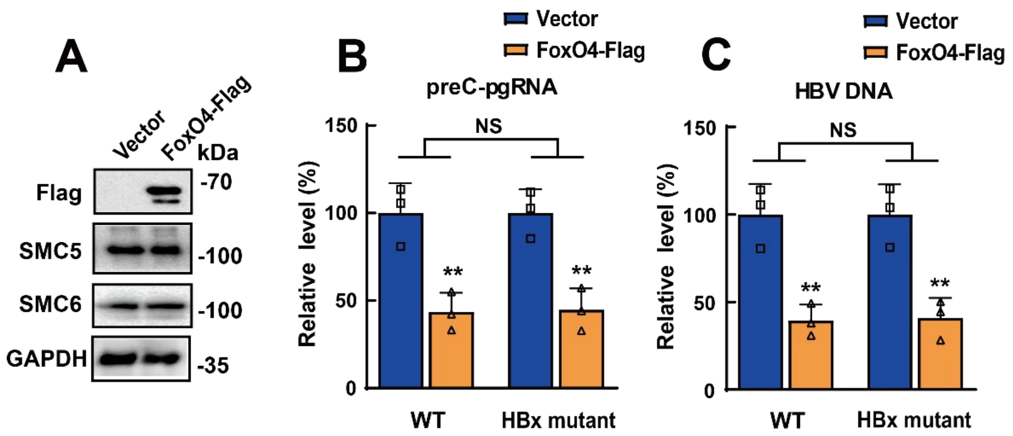

Supporting FIG. S6. FoxO4 fails to upregulate the expression level of SMC5 and SMC6 and inhibits the rcccDNA-driven transcription and HBV replication independent of HBx. **A:** FoxO4-Flag or control empty vector was transfected into Huh7 cells together with prcccDNA/Cre plasmids. 24 hours posttransfection, the protein level of FoxO4, SMC5 and SMC6 were examined by Western blot. **B, C:** FoxO4-Flag was transfected into Huh7 cells together with prcccDNA/Cre (WT) or prcccDNA with the HBx mutated/Cre (HBx mutant). 48 hours posttransfection, levels of preC-pgRNA (B) and HBV DNA (C) were determined by qRT-PCR and qPCR, respectively. Data are shown as means ± SD of triplicates and are representative of three independent experiments. $^{**}P < 0.01$. NS, no significance.

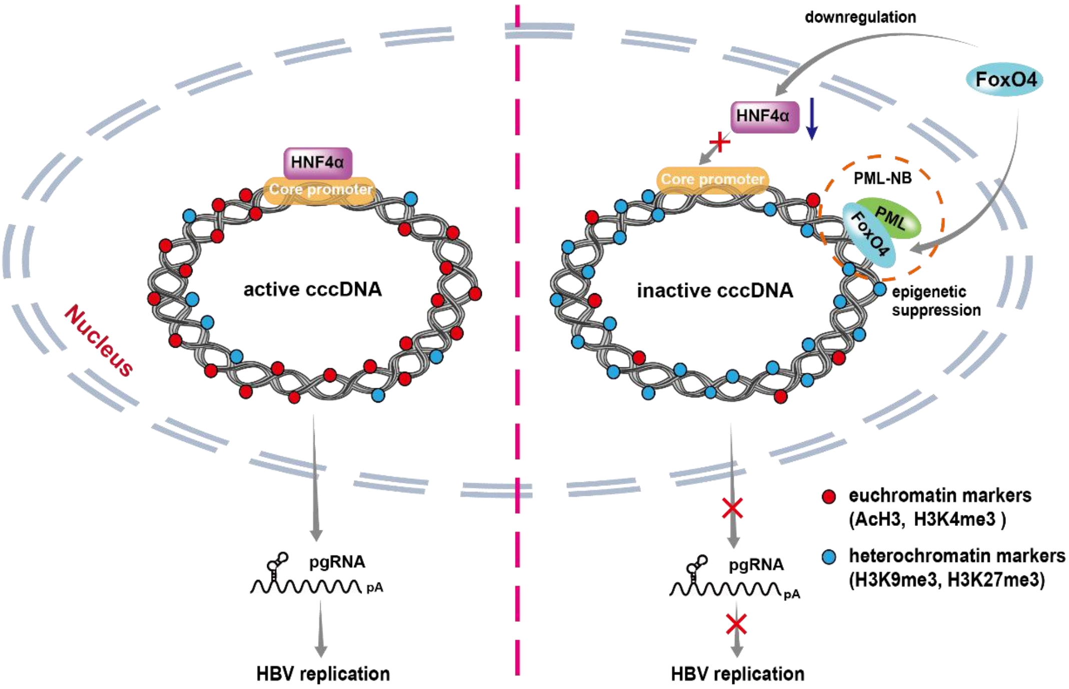

Supporting FIG. S7. Graphical illustration of FoxO4-mediated inhibitory effect on HBV cccDNA. FoxO4 displays inhibitory effect on cccDNA-driven transcription and HBV replication, but it does not affect the level of cccDNA itself. Mechanistically, FoxO4 expression could lead to the epigenetic suppression of cccDNA through co-localizing with PML in the nuclear bodies and interacting with PML. Downregulation of PML significantly attenuates FoxO4-mediated epigenetic suppression of cccDNA and the following cccDNA transcription and HBV production. On the other hand, FoxO4 expression leads to the downregulation of HNF4α. However, HNF4α appears not to be involved in FoxO4-mediated epigenetic suppression of cccDNA, although it contributes indeed to FoxO4 inhibition of HBV core promoter activity. Together, FoxO4 might inhibit cccDNA transcription and HBV replication via a two-part mechanism: one is epigenetic suppression of cccDNA via interacting with PML, the other is inhibition of HBV core promoter activity involving the genetic downregulation of HNF4α.

Supplemental Table 1: Primers/siRNA oligos

HBV RNAs	Forward	5'-GCACTTCGCTTCACCTCTGC-3'
	Reverse	5'-CTCAAGGTCGGTCGTTGACA-3'
preC-pgRNA	Forward	5'-TGTTCAAGCCTCCAAGCT-3'
	Reverse	5'-GGAAAGAAGTCAGAAGGCAA-3'
HBV DNA	Forward	5'-CCCGTTTGTCCTCTAATTCC-3'
	Reverse	5-GTCCGAAGGTTTGGTACAGC-3'
HBV cccDNA	Forward	5′-CTCCCCGTCTGTGCCTTCT-3′
	Reverse	5′-GCCCCAAAGCCACCCAAG-3′
HBV rcccDNA	Forward	5′-CAAGACAGGTTTAAGGAGAC-3 ′
	Reverse	5′-GAGAGAAAGGCAAAGTGGAT-3′
β-globin	Forward	5′-GTGCACCTGACTCCTGAGGAGA-3′
	Reverse	5′-CCTTGATACCAACCTGCCCAG-3′
GAPDH	Forward	5′-GCCTCTGCGCCCTTGAGCTA-3′
	Reverse	5′-GATGCGGCCGTCTCTGGAAC-3′
IFN-β	Forward	5′-GACCAACAAGTGTCTCCTCCAAA-3′
	Reverse	5′-GAACTGCTGCAGCTGCTTAATC-3′
OAS1	Forward	5′-TCCACCTGCTTCACAGAACTACA-3′
	Reverse	5′-TGGGCTGTGTTGAAATGTGTTT-3′
ISG56	Forward	5′-GCCTTGCTGAAGTGTGGAGGAA-3′
	Reverse	5′-ATCCAGGCGATAGGCAGAGATC-3′
FoxO4 siRNA#1	Sense	5′-CGCGAUCAUAGACCUAGAUTT-3′
	Anti-sense	5′-AUCUAGGUCUAUGAUCGCGTT-3′
FoxO4 siRNA#2	Sense	5’-GUGACAUGGAUAACAUCAUTT-3’
	Anti-sense	5’-AUGAUGUUAUCCAUGUCACTT-3’
PML siRNA	Sense	5′-AGAUGCAGCUGUAUCCAAG-3′
	Anti-sense	5′-CUUGGAUACAGCUGCAUCU-3′

Supplemental Table 2: Antibodies

Anti-Flag	Bioworld Biotechnology, AP0007
GAPDH	Cell Signaling Technology, #8884
FoxO4	Cell Signaling Technology, #9472
HBcAg	Abcam, ab8639
H3	Santa Cruz, sc-8654
AcH3	Cell Signaling Technology, #9677
H3K4me3	Cell Signaling Technology, #9727
H3K9me3	Cell Signaling Technology, #5327
H3K27me3	Cell Signaling Technology, #9733
Anti-HA	Bioworld Biotechnology, AP0005
PML	Cell Signaling Technology, #33156
p53	Cell Signaling Technology, #2524
HNF4α	Bioworld Biotechnology, BS6888
SMC5	Abcam, ab154103
SMC6	Abcam, ab155495

Supplemental Table S3 Characteristics of CHB patients and Control individuals

	No.	Age (yr)	Gender	HBsAg	Serum HBV-DNA (IU/mL)	Serum AST (IU/L)	Serum ALT (IU/L)		No.	Age (yr)	Gender	HBsAg	Serum HBV-DNA (IU/mL)	Serum AST (IU/L)	Serum ALT (IU/L)
**CHB**	1	38	M	+	1.91E+05	46	49	**Control**	1	38	M	—	—	26	29
**patients**	2	21	M	+	3.30E+03	25	38	**individuals**	2	45	F	—	—	12	21
	3	36	M	+	7.09E+07	60	87		3	51	M	—	—	32	70
	4	46	M	+	1.69E+03	18	27		4	39	M	—	—	23	25
	5	32	F	+	6.76E+05	48	103		5	52	M	—	—	17	21
	6	58	F	+	4.18E+03	24	23		6	37	M	—	—	15	11
	7	35	M	+	1.91E+04	39	108		7	46	F	—	—	17	25
	8	23	F	+	3.68E+06	18	17		8	24	F	—	—	26	35
	9	52	M	+	1.07E+05	30	60		9	61	M	—	—	11	19
	10	39	M	+	9.87E+03	29	82		10	38	M	—	—	32	39
	11	54	M	+	8.76E+02	23	23		11	31	M	—	—	21	28
	12	53	M	+	1.01E+03	29	30		12	45	F	—	—	33	29
	13	45	F	+	1.01E+03	18	22		13	27	M	—	—	23	23
	14	37	F	+	5.56E+05	51	57		14	34	M	—	—	26	40
	15	36	M	+	4.48E+01	60	87		15	37	F	—	—	36	42
	16	44	M	+	9.09E+04	34	27		16	39	M	—	—	20	28
	17	37	F	+	6.68E+05	31	34		17	41	F	—	—	30	38
	18	39	M	+	5.68E+08	42	48		18	29	M	—	—	19	25
